# Lipopolysaccharide Diversity Evolving in *Helicobacter pylori* Communities through Genetic Modifications in Fucosyltransferases

**DOI:** 10.1371/journal.pone.0003811

**Published:** 2008-11-26

**Authors:** Christina Nilsson, Anna Skoglund, Anthony P. Moran, Heidi Annuk, Lars Engstrand, Staffan Normark

**Affiliations:** 1 Department of Microbiology, Tumor and Cell Biology, Karolinska Institutet, Stockholm, Sweden; 2 Swedish Institute for Infectious Disease Control, Solna, Sweden; 3 Department of Microbiology, School of Natural Sciences, National University of Ireland, Galway, Ireland, United Kingdom; 4 Institute for Glycomics, Gold Coast Campus, Griffith University, Queensland, Australia; University of British Columbia, Canada

## Abstract

*Helicobacter pylori* persistently colonizes the gastric mucosa of half the human population. It is one of the most genetically diverse bacterial organisms and subvariants are continuously emerging within an *H. pylori* population. In this study we characterized a number of single-colony isolates from *H. pylori* communities in various environmental settings, namely persistent human gastric infection, *in vitro* bacterial subcultures on agar medium, and experimental *in vivo* infection in mice. The lipopolysaccharide (LPS) O-antigen chain revealed considerable phenotypic diversity between individual cells in the studied bacterial communities, as demonstrated by size variable O-antigen chains and different levels of Lewis glycosylation. Absence of high-molecular-weight O-antigen chains was notable in a number of experimentally passaged isolates *in vitro* and *in vivo*. This phenotype was not evident in bacteria obtained from a human gastric biopsy, where all cells expressed high-molecular-weight O-antigen chains, which thus may be the preferred phenotype for *H. pylori* colonizing human gastric mucosa. Genotypic variability was monitored in the two genes encoding α1,3-fucosyltransferases, *futA* and *futB*, that are involved in Lewis antigen expression. Genetic modifications that could be attributable to recombination events within and between the two genes were commonly detected and created a diversity, which together with phase variation, contributed to divergent LPS expression. Our data suggest that the surrounding environment imposes a selective pressure on *H. pylori* to express certain LPS phenotypes. Thus, the milieu in a host will select for bacterial variants with particular characteristics that facilitate adaptation and survival in the gastric mucosa of that individual, and will shape the bacterial community structure.

## Introduction

*Helicobacter pylori* infects the human gastric mucosa, primarily during early childhood, and establishes a lifelong persistence within its host [1], [2]. An estimated half of the human population carries this bacterium, but most individuals never perceive its presence and will remain clinically asymptomatic throughout their life span. However, in response to the bacterial presence in the stomach, a minority of infected individuals will develop disease, such as peptic ulcer or gastric cancer [3], [4]. Disease development is a consequence of the interplay between bacterial, host and environmental factors. One such bacterial virulence-associated trait is the *cag* pathogenicity island (PAI), a transposable genetic element that encodes a type IV secretion system. *H. pylori* cells that express this element induce pathological alterations in the gastric mucosa, thereby promoting disease development [5].

*H. pylori* is an exceptionally diverse bacterial species and isolates that colonize unrelated individuals are genetically distinct [6], [7]. Although infection by multiple strains has been described [8], [9], *H. pylori* cells within an individual are commonly genetically similar, suggesting that they stem from a single infecting strain [10]–[12]. Nevertheless, within such a confined bacterial community, individual subclones are not entirely identical, but display divergence through subtle genetic and, consequently, phenotypic variants [13]–[19]. This marked genetic diversity in *H. pylori* is attributable to both a high incidence of point mutations as well as remarkably frequent homologous recombination events in the genome [13], [18], [20].

**Figure 1:**
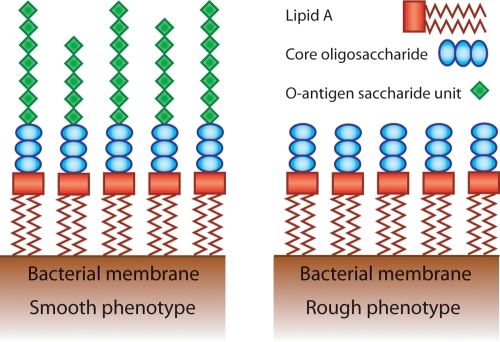
Schematic representation of the LPS molecule. The lipid A moiety consist of two parts, a lipid part composed by fatty acid chains that anchors LPS in the outer membrane and a sugar backbone that links to the core oligosaccharide. The outermost region of the LPS is built of repetitive saccharide units that form the O-antigen chain. The number of such units on a LPS molecule may vary within and between bacterial cells. In *H. pylori* these O-antigen saccharide units are usually structurally identical to Lewis antigens. For a detailed structure of *H. pylori* LPS, refer to references [21] and [32]. Bacteria that display the smooth LPS phenotype express all three moieties of the LPS molecule (left panel) whereas rough bacteria have lost the expression of the O-antigen chains (right panel).

Lipopolysaccharide (LPS) is a main component of the outer membrane of Gram negative bacteria. A typical LPS molecule is composed of three parts; a lipid A moiety that anchors in the lipid bilayer, a conserved saccharide core region, and a variable saccharide O-antigen chain (Figure 1) [21]. In *H. pylori*, the appearance of the LPS can vary extensively between different isolates [22], [23]. The smooth phenotype that is usually found in clinical isolates exhibit high-molecular weight LPS and carries extended O-antigen chains, built of repetitive carbohydrate units. Conversely, some strains lack expression of the O-antigen chain, presenting a rough phenotype (Figure 1) [22]. This is a characteristic of many laboratory strains and it has been shown that, influenced by growth conditions, strains can reversibly switch between the smooth- and rough-LPS phenotype [22], [24].

One unique feature of the O-antigen chain in *H. pylori* is its display of Lewis antigens, structures that are also found on the surface of certain human cells, such as erythrocytes and epithelial cells [25]. Lewis antigens are classified into two groups, type 1 and type 2, reflecting a structural variation in the core precursor. The LPSs of *H. pylori* strains are commonly glycosylated with the type 2 antigens Lewis x (Le^x^) and/or Le^y^, while the corresponding type 1 antigens, Le^a^ and Le^b^, as well as other related Lewis antigens, are observed at lower frequencies [26]–[32]. A typical *H. pylori* O-antigen chain is glycosylated with multiple internal Le^x^ units and possesses either Le^x^ or Le^y^ at the terminal position. Since the initial discovery of Le^x^ in the LPS of *H. pylori* [33], a number of different biological functions have been attributed to the presence of Lewis antigens on the surface of the bacterium. These proposed roles include; promoting adhesion and colonization, escape host recognition by molecular mimicry, modulation of the host response through interaction with immune cells, and induction of gastric autoimmunity (reviewed in [32]). Although there are experimental data in support of each of these roles, conflicting data have also been presented, and much remains to be revealed before we fully recognize the complex biological potential of this surface molecule.

**Figure 2:**
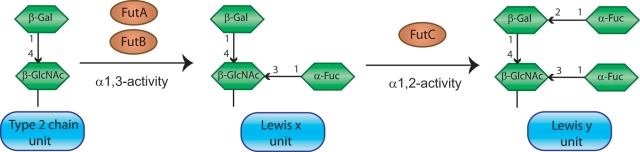
Structures and synthetic pathway for Lewis antigens in *H. pylori*. FutA and FutB can fucosylate Type 2 chains that are present in the elongating LPS molecule via an α1,3-FucT activity, creating the Le^x^ antigen. Repetitive copies of such Le^x^ units are usually present in the O-antigen chain of *H. pylori*. FutC that possesses an α1,2-FucT activity may subsequently add a second fucose to the Le^x^ structure, generating the Le^y^ antigen. This process will cap the O-antigen chain and block further elongation of the LPS molecule and, consequently, Le^y^ can only be found at the terminal position of the O-antigen chain. Type 1 chains are structurally similar to the Type 2 chains and only differ in that they possess a β1,3-linkage instead of a β1,4-linkage between the Gal and GlcNAc saccharides in the precursor unit. In this case, fucosylation by FutA and FutB will require an α1,4-FucT activity and create a Le^a^ antigen, prior to α1,2-fucosylation by FutC that produces a di-fucosylated Le^b^ antigen. Gal = galactose, GlcNAc = N-Acetylglucosamine, Fuc = fucose.

Synthesis of Lewis antigens involves the transfer of fucose residues to a carbohydrate core precursor, a reaction catalyzed by fucosyltransferases (FucTs) (Figure 2). *H. pylori* contains three genes coding for FucTs; *futA*, *futB* and *futC*. FutA and FutB are paralogous and can have either α1,3- and/or α1,4-FucT activity, thus acting on type 2 (synthesizing Le^x^) and/or type 1 (synthesizing Le^a^) precursors, respectively. FutC is an α1,2-FucT that adds a second fucose residue to Le^x^ or Le^a^ to create difucoslylated Le^y^ or Le^b^ [32], [34]. The *H. pylori* FucTs are similar to their mammalian counterparts in the catalytic region, where they share weak homology, but differ in that they do not contain a transmembrane domain and cytosolic tail in the N-terminal part [34], [35]. However, *H. pylori* α1,3/4-FucTs harbor a unique heptad-repeat region that is not present in other FucTs. This domain, together with the adjacent C-terminal amphipatic α-helices, may be functionally equivalent to the N-terminal stem region and transmembrane domain of corresponding mammalian FucTs [34], [36].

Although the immense genetic diversity of *H. pylori* is often portrayed as presence or absence of specific genes [37], [38], all three genes encoding FucTs appear to be universally present in every isolate that exhibit Lewis antigen mimicry [39]. Nevertheless, the activity of these enzymes may differ considerably between isolates. All three FucTs are subjected to translational frame-shifting in homopolymeric tracts within the genes, thereby causing the enzymes to switch between an active and silent stage [40], [41]. The specificity of FutA and FutB is, moreover, directed by the nature of a short variable domain that dictates the enzymes towards either α1,3- and/or α1,4-FucT activity [36], [42]. The enzymatic activity of FutA and FutB is also affected by diversity in the heptad-repeat region. This domain functions as a molecular ruler, and the number of repeat units controls the enzymatic activity, such that O-antigen chains of different lengths become fucosylated [43]. Collectively, this enzymatic diversity in FucTs creates variations in the LPS phenotypes that are distinctive between different *H. pylori* strains.

In addition to the vast diversity of LPS structures between *H. pylori* strains, there is also apparent micro-diversity within a population of cells [43]–[45]. By varying the appearance of LPS as a surface molecule, persistence and pathogenicity may be significantly affected. Diversity in LPS molecules, thus, may represent a critical means for bacterial influence on the outcome of infection. In the present investigation, we perform an in-depth study of phenotypic switching in LPS moieties within *H. pylori* communities. The aim was to assess the level of LPS diversity that exist between closely related *H. pylori* cells in a persistent human infection and to monitor the diversification process that follows a strain during experimental *in vitro* and *in vivo* passages. We moreover describe how such diversity correlates to genetic mosaics in corresponding FucTs.

## Results

### Diversity within an *H. pylori* community in a persistent human infection

**Figure 3:**
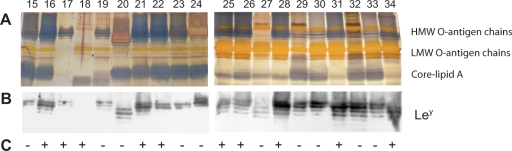
LPS profiles of 20 intra-patient single-colony *H. pylori* isolates. (A) Silver staining of extracted LPS from subisolates 67:15–34, illustrating the diversity of O-antigen chains within an *H. pylori* community in a persistent infection. The migration of high-molecular-weight (HMW) O-antigen chains, low-molecular-weight (LMW) O-antigen chains and core-lipid A is indicated. (B) Immunoblot analysis with antibodies detecting Le^y^ reveals that all but one isolate express this antigen, but that the amount and size of Le^y^-bearing O-antigen chains differ among isolates. The area included in the immunoblot of part B corresponds to the HMW and LMW O-antigen regions in the silver stained gel of part A. (C) *cag* PAI status, as analyzed by PCR, is shown as + (present) or − (absent).

We investigated the intra-population diversity of *H. pylori* cells that colonized the stomach of a 90-year old patient. Genomic fingerprinting of twenty subisolates, by means of arbitrary primed PCR, showed indistinguishable patterns (data not shown), implicating a common origin. Silver staining of purified LPS from the same isolates revealed that the bacterial community comprised a mixture of cells with variations in the typical ladder pattern, representing the lengths of the O-antigen chains (Figure 3A). In immunoblot experiments, all but one isolate (67:18) reacted distinctively with the Le^y^ antibody, although the amount and size of glycosylated O-antigen chains varied significantly between the different isolates (Figure 3B). None of the analyzed samples revealed any detectable levels of Le^x^ (data not shown). Thus, the *H. pylori* population in this chronically infected patient, was focused towards simultaneous expression of both α1,3- and α1,2-FucTs.

In addition to the LPS phenotypic diversity, PCR analyses of the *cagA* gene and the *cag* PAI empty site revealed that the *H. pylori* population that colonized this individual also displayed diversity in this genetic region. Half (10/20) of the isolates possessed the *cag* PAI whereas the remaining isolates were negative for this locus (Figure 3C). It has previously been suggested that expression of Lewis antigens correlates to presence of the *cagA* gene [27], [46]. In our sampling of intra-patient isolates, all but one isolate was Le^y^-positive, and we could see no relationship between *cag* status and Lewis expression. The *cag* PAI of *H. pylori* is required for the bacterial stimulation of host cells to produce interleukin-8 (IL-8). As expected, a strong correlation between *cag* PAI status, but not Lewis antigen status, and the ability to induce secretion of IL-8 in cultured epithelial AGS cells was observed in the studied subclones (data not shown).

### Altered LPS phenotype following *in vitro* subculturing

We sought to explore the stability of *H. pylori* LPS structure and the effect of successive *in vitro* propagation on its appearance. Two subclones were selected from the population of bacteria that colonized the above-described patient. These two isolates, 67:20 and 67:21, were both Le^y^-positive but differed in their displayed LPS in that 67:20 produced Le^y^-decorated O-antigen chains of a lower molecular weight than those observed in 67:21 (Figure 3). The isolates were subjected to 50 passages of subculturing on agar medium using two different approaches; transfer by either a sweep of bacteria or by a single-cell colony in each step, creating passages with large (50pL) and small (50pS) bottlenecks, respectively.

After 50 large bottleneck *in vitro* passages of 67:20 and 67:21, the LPS profiles of the recovered bacterial population appeared unaffected and O-antigen chain length as well as Lewis glycosylation of the subcultured isolates were indistinguishable to the corresponding original wild-type strains (data not shown). Furthermore, smooth-LPS profiles were identical both with and without a final subculture in broth (data not shown), indicating that the strains did not lose the production of the O-antigen chain during the repeated passages on agar medium in this study, as can sometimes occur [22], [24].

**Figure 4 pone-0003811-g004:**
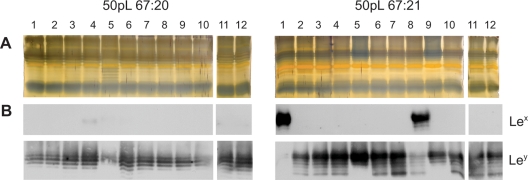
LPS profiles of *H. pylori* isolates after large bottleneck *in vitro* passages (50pL). Silver staining (A) and Immunoblot analysis, detecting Le^x^ and Le^y^ (B), of extracted LPS from twelve single-colony isolates per strain, obtained after 50 *in vitro* passages of bacterial sweeps on agar medium, reveals minor differences in the LPS molecules.

Subculturing with a large bottleneck may, however, generate a diverse mixture of bacterial cells with divergent traits within the bacterial community. In order to study the phenotypic appearance of individual cells and the diversity within the subcultured populations, twelve single-cell colonies were isolated from each pool of bacteria after 50 *in vitro* passages of 67:20 and 67:21, respectively. Patterns of silver-stained O-antigen chains from the single-colony derived isolates revealed no major differences to the original wild-type strains. All isolates expressed smooth-LPS and the size difference between the prominent high-molecular-weight O-antigen chains in 67:20- and 67:21-derived isolates was apparent in all cases (Figures 4A and S1). All 67:20-derived isolates expressed Le^y^ on O-antigen chains of an equivalent size as those observed in the original strain. Although most isolates derived from the population of subcultured 67:21 bore Le^y^-decorated O-antigen chains, phenotypically similar to strain 67:21, two of the twelve subisolates (50pL 67:21-1 and -8) also expressed considerable levels of Le^x^. Both isolates retained Le^y^ expression, but at lower levels than the remaining isolates, and in the case of 50pL 67:21-1, only traces of Le^y^ was detected by immunoblotting (Figure 4B), suggesting a reduced expression of FutC.

**Figure 5 pone-0003811-g005:**
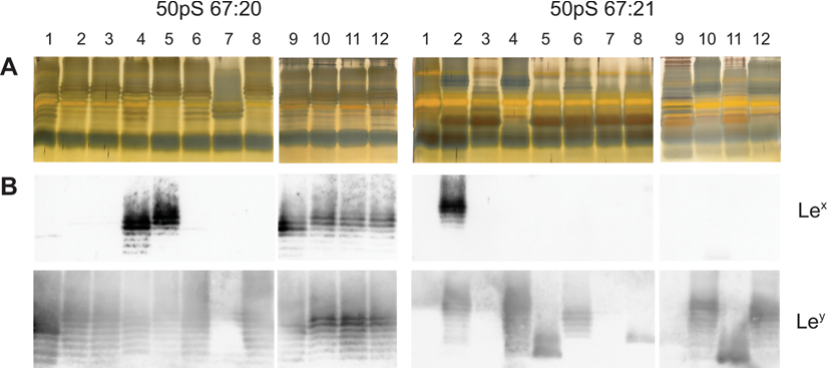
LPS profiles of *H. pylori* isolates after small bottleneck *in vitro* passages (50pS). Twelve colonies from 67:20 and 67:21 were initially isolated and passaged in parallel for 50 times on agar medium with one single colony being transferred in each subculturing step. Silver staining (A) and Immunoblot analysis, detecting Le^x^ and Le^y^ (B), of extracted LPS shows large variability in the O-antigen expression after such propagation, with loss of prominent high-molecular weight O-antigens in several of the 67:21-derived isolates..

For studies of small bottleneck *in vitro* passages, we maintained twelve lineages each of 67:20 and 67:21 with a limit of one single-cell colony in each transfer. After 50 successive subculturing steps on agar medium and a final subculture in broth, numerous isolates revealed a different phenotype than the original wild-type strains 67:20 and 67:21. Silver-stained LPS molecules displayed substantial pattern differences between isolates. High-molecular-weight O-antigen chains were of various sizes in both 67:20 and 67:21-derived isolates. Notably, in the case of 67:21, only four isolates (50pS 67:21-2, -4, -10 and -12) expressed high-molecular-weight polymers of similar phenotype as the original 67:21 isolate, while the remaining eight isolates showed evidence of a reduced O-antigen chain length (Figures 5A and S1). Immunoblot experiments showed that six isolates derived from 67:20, in addition to Le^y^, simultaneously expressed Le^x^. The same was true for one 67:21-derived isolate. Moreover, the sizes of major Le^x^- and Le^y^-glycosylated O-antigens differed between the recovered isolates. Most prominent was the detection of only low-molecular-weight Le^y^-glycosylation in three 67:21-derived isolates (50pS 67:21-5, -8 and -11), all of which displayed diminished levels of high-molecular-weight O-antigen chains in the silver staining experiment. Another four isolates with similar low-molecular-weight O-antigen chain phenotypes (50pS 67:21-1, -3, -7 and -9) revealed no detectable amounts of either Le^x^ or Le^y^ (Figure 5B).

### Instability in the LPS molecule following experimental *in vivo* infection

**Figure 6:**
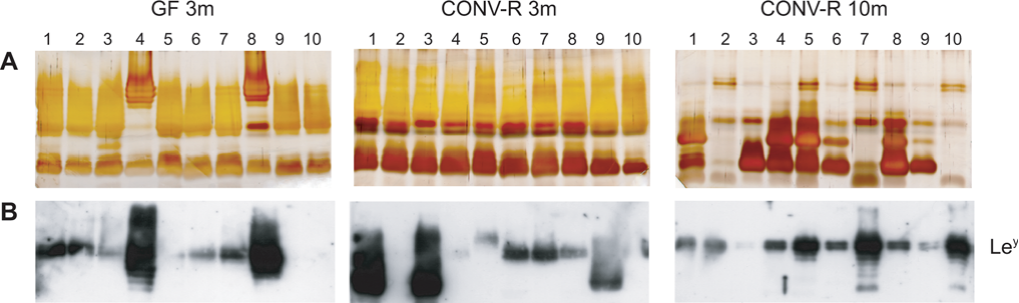
LPS profiles of *H. pylori* isolates after *in vivo* passage in mice. Strain 67:21 was used to infect one germ-free mouse for 3 months (GF 3m) and two conventionally raised mice for three (CONV-R 3m) and ten (CONV-R 10m) months, respectively. LPS was analyzed by silver staining (A) and immunoblot with antibodies detecting Le^y^ (B) from ten emerging isolates from each mouse. The analyses revealed that the majority of isolates expressed LPS molecules with reduced levels of Le^y^-decorated high-molecular-weight O-antigens, as compared to the wildtype 67:21 strain.

An animal model was used to propagate bacteria *in vivo* and study LPS diversification after transfer to a new host. Transgenic mice, expressing Le^b^ on gastric epithelial cells that act as a receptor for *H. pylori*, were inoculated with strain 67:21 [15], [47]. Ten single-cell colonies were isolated from each of three mice, one ex-germ-free mouse, mono-associated with 67:21 for three months (GF 3m), and two conventionally raised mice harboring a normal gastrointestinal flora, infected for three (CONV-R 3m) and ten (CONV-R 10m) months, respectively. Silver staining of LPS preparations from the thirty emerging isolates revealed that only two isolates (GF 3m-4 and -8) produced O-antigen chains with a similar profile as the original 67:21 isolate (Figures 6A and S1). The other isolates lacked the distinct high-molecular-weight ladder pattern and, rather, produced glycolipids with more pronounced low-molecular-weight structures. Immunoblot experiments further supported this observation, since only the two above-mentioned isolates exhibited patterns similar to the original isolate with prominent high-molecular-weight Le^y^-glycosylation (Figure 6B). The majority of isolates displayed a more restricted glycosylation with typically only one O-antigen chain-size bearing the Le^y^ antigen. Three isolates (CONV-R 3m-1, -3 and -9) preferentially expressed Le^y^-glycosylated O-antigen chains of a lower molecular weight. Three of the isolates (GF 3m-9, -10 and CONV-R 3m-2) did not show any detectable amounts of Le^y^-expression, and an additional number of isolates displayed only very weak amounts of this antigen in immunoblot experiments (Figure 6B). Moreover, one of the recovered isolates (CONV-R 10m-2) expressed weak levels of Le^x^ (data not shown).

### Genetic exchange between *futA* and *futB*

**Figure 7:**
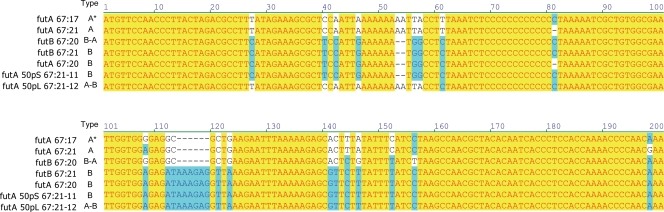
Genetic exchange between *futA* and *futB* in *H. pylori* communities. The 5′-region of *futA* and *futB* was sequenced in all isolates included in this study. The figure illustrates representative isolates that covers all nucleotide differences that were observed, the only exception being insertion/deletions of C-residues in the homopolymeric tract that varied according to table 1–4.

In order to link the observed phenotypic diversity of Lewis expression to genetic alterations, we examined the genetic background of the two *H. pylori* α1,3-FucTs in all isolates. When comparing the first 200 bp of the 5′-region of *futA* and *futB* genes, it was apparent that the gene content of these two homologues was dynamic. In the studied population of single-colony isolates that colonized the 90-year old patient, most isolates contained the same alleles of *futA* and *futB* at these two loci (type A and B, Figure 7, Table 1). However, two out of 20 subisolates (67:20 and 67:27) showed evidence of recombination between *futA* and *futB*. In these isolates the corresponding *futA* sequence was detected at the *futB* locus whereas the *futA* locus contained a combination of *futB* and *futA* sequences (type B-A, Figure 7, Table 1). This phenomenon of genetic exchange between the two *fut* genes was also found to occur during subculturing *in vitro*. After passage with a large bottleneck, the *futA* locus of all 67:21-derived isolates (50pL 67:21) contained a hybrid of the parental *futA* and *futB* sequences (type A-B, Figure 7, Table 2). Moreover, one of the twelve small bottleneck subcultured 67:21-lineages (50pS 67:21-11) possessed the *futB* allele at both loci (Figure 7, Table 3). Analyses of the same regions in isolates obtained after *in vivo* passage of 67:21 in mice did not show signs of recombination, and all subisolates bore the same alleles as the original isolate (Table 4).

**Table 1:** *futA* and *futB* genotypes in an *H. pylori* population colonizing human gastric mucosa.

	*futA*			*futB*		
Subisolate	Type^1^	C-tract^2^	Heptads^3^	Type	C-tract	Heptads
67:15	A	13 −	2	B	13 +	8
67:16	A	12 −	2	B	13 +	8
67:17	A*	13 −	2	B	13 +	8
67:18	A*	14 +	5	B	13 +	7
67:19	A*	13 −	2	B	13 +	8
67:20	B	12 −	17	B-A	13 +	5
67:21	A	12 −	2	B	13 +	8
67:22	A	12 −	2	B	13 +	8
67:23	A	13 −	2	B	13 +	8
67:24	A	13 −	2	B	13 +	8
67:25	A	12 −	2	B	13 +	8
67:26	A	12 −	2	B	13 +	8
67:27	B	12 −	15	B-A	13 +	5
67:28	A	12 −	2	B	13 +	8
67:29	A	13 −	2	B	13 +	8
67:30	A	13 −	2	B	13 +	8
67:31	A	12 −	2	B	13 +	8
67:32	A	13 −	2	B	13 +	8
67:33	A	13 −	2	B	13 +	8
67:34	A	12 −	2	B	13 +	7

1 *fut* allele at locus, based on sequencing of 200 bp at the 5′ of gene. The types A and B are referred to the genetic sequence present at the corresponding locus in strain 67:21. A^*^ differs from A at two nucleotide positions, according to Figure 7.

2 Number of C-residues in 5′ homopolymeric tract and reading frame of the gene (+ in frame, − out of frame)

3 Number of C-terminal heptad-repeats

**Table 2 pone-0003811-t002:** *futA* and *futB* genotypes of subisolates after 50 *in vitro* passages with a large bottleneck.

	*futA*			*futB*		
Subisolate	Type^1^	C-tract^2^	Heptads^3^	Type	C-tract	Heptads
50pL 67:20-1	B	12 −	17	B-A	13 +	5
50pL 67:20-2	B	12 −	17	B-A	13 +	5
50pL 67:20-3	B	12 −	17	B-A	13 +	5
50pL 67:20-4	B	12 −	17	B-A	13 +	5
50pL 67:20-5	B	12 −	17	B-A	13 +	5
50pL 67:20-6	B	12 −	17	B-A	13 +	5
50pL 67:20-7	B	12 −	17	B-A	13 +	5
50pL 67:20-8	B	12 −	17	B-A	13 +	5
50pL 67:20-9	B	12 −	17	B-A	13 +	5
50pL 67:20-10	B	12 −	17	B-A	13 +	5
50pL 67:20-11	B	12 −	17	B-A	13 +	5
50pL 67:20-12	B	12 −	17	B-A	13 +	5
50pL 67:21-1	ND	ND	ND	ND	ND	ND
50pL 67:21-2	A-B	13 −	2	B	13 +	8
50pL 67:21-3	A-B	13 −	2	B	13 +	8
50pL 67:21-4	A-B	13 −	2	B	13 +	8
50pL 67:21-5	A-B	12 −	2	B	13 +	8
50pL 67:21-6	A-B	13 −	2	B	13 +	8
50pL 67:21-7	A-B	13 −	2	B	13 +	8
50pL 67:21-8	A-B	13 −	2	B	13 +	8
50pL 67:21-9	A-B	12 −	2	B	13 +	8
50pL 67:21-10	A-B	13 −	2	B	13 +	8
50pL 67:21-11	A-B	13 −	2	B	13 +	8
50pL 67:21-12	A-B	13 −	2	B	13 +	8

1 *fut* allele at locus, based on sequencing of 200 bp at the 5′ of gene. The types A and B are referred to the genetic sequence present at the corresponding locus in strain 67:21.

2 Number of C-residues in 5′ homopolymeric tract and reading frame of the gene (+ in frame, − out of frame)

3 Number of C-terminal heptad-repeats

ND: not determined

**Table 3 pone-0003811-t003:** *futA* and *futB* genotypes of subisolates after 50 *in vitro* passages with a small bottleneck.

	*futA*			*futB*		
Subisolate	Type^1^	C-tract^2^	Heptads^3^	Type	C-tract	Heptads
50pS 67:20-1	B	12 −	17	B-A	13 +	3
50pS 67:20-2	B	12 −	17	B-A	13 +	5
50pS 67:20-3	B	12 −	17	B-A	13 +	5
50pS 67:20-4	B	12 −	17	B-A	13 +	4
50pS 67:20-5	B	12 −	17	B-A	13 +	5
50pS 67:20-6	B	12 −	17	B-A	13 +	4
50pS 67:20-7	B	12 −	17	B-A	12 −	5
50pS 67:20-8	B	12 −	17	B-A	13 +	5
50pS 67:20-9	B	12 −	17	B-A	13 +	4
50pS 67:20-10	B	12 −	17	B-A	13 +	5
50pS 67:20-11	B	13 +	17	B-A	13 +	5
50pS 67:20-12	B	12 −	17	B-A	13 +	5
50pS 67:21-1	A	12 −	2	B	12 −	8
50pS 67:21-2	A	12 −	2	B	13 +	8
50pS 67:21-3	A	12 −	2	B	11 −	8
50pS 67:21-4	A	12 −	2	B	13 +	8
50pS 67:21-5	A	12 −	2	B	13 +	7
50pS 67:21-6	A	12 −	2	B	13 +	6
50pS 67:21-7	A	12 −	2	B	11 −	8
50pS 67:21-8	A	13 −	2	B	12 −	8
50pS 67:21-9	A	12 −	2	B	12 −	8
50pS 67:21-10	A	12 −	2	B	13 +	8
50pS 67:21-11	B	13 +	2	B	13 +	8
50pS 67:21-12	A	12 −	2	B	13 +	7

1 *fut* allele at locus, based on sequencing of 200 bp at the 5′ of gene. The types A and B are referred to the genetic sequence present at the corresponding locus in strain 67:21.

2 Number of C-residues in 5′ homopolymeric tract and reading frame of the gene (+ in frame, − out of frame)

3 Number of C-terminal heptad-repeats

**Table 4:** *futA* and *futB* genotypes of subisolates after *in vivo* passages in mice.

	*futA*			*futB*		
Subisolate	Type^1^	C-tract^2^	Heptads^3^	Type	C-tract	Heptads
GF 3m-1	A	12 −	2	B	13 +	8
GF 3m-2	A	12 −	2	B	13 +	8
GF 3m-3	A	12 −	2	B	13 +	8
GF 3m-4	A	12 −	2	B	13 +	8
GF 3m-5	A	12 −	2	B	13 +	8
GF 3m-6	A	12 −	2	B	13 +	8
GF 3m-7	A	12 −	2	B	13 +	8
GF 3m-8	A	12 −	2	B	13 +	8
GF 3m-9	A	12 −	2	B	13 +	8
GF 3m-10	A	12 −	2	B	13 +	8
CONV-R 3m-1	A	12 −	2	B	13 +	4
CONV-R 3m-2	A	12 −	2	B	13 +	8
CONV-R 3m-3	A	12 −	2	B	13 +	4
CONV-R 3m-4	A	12 −	2	B	13 +	8
CONV-R 3m-5	A	12 −	2	B	13 +	8
CONV-R 3m-6	A	13 −	2	B	13 +	8
CONV-R 3m-7	A	12 −	2	B	13 +	8
CONV-R 3m-8	A	13 −	2	B	13 +	8
CONV-R 3m-9	A	12 −	2	B	13 +	4
CONV-R 3m-10	A	12 −	2	B	13 +	8
CONV-R 10m-1	A	12 −	2	B	13 +	8
CONV-R 10m-2	A	12 −	2	B	13 +	8
CONV-R 10m-3	A	12 −	2	ND	ND	8
CONV-R 10m-4	A	12 −	2	B	13 +	8
CONV-R 10m-5	A	12 −	2	B	13 +	8
CONV-R 10m-6	A	12 −	2	B	13 +	8
CONV-R 10m-7	A	12 −	2	B	13 +	8
CONV-R 10m-8	A	12 −	2	ND	ND	8
CONV-R 10m-9	ND	ND	2	B	13 +	8
CONV-R 10m-10	A	12 −	2	B	13 +	8

1 *fut* allele at locus, based on sequencing of 200 bp at the 5′ of gene. The types A and B are referred to the genetic sequence present at the corresponding locus in strain 67:21.

2 Number of C-residues in 5′ homopolymeric tract and reading frame of the gene (+ in frame, − out of frame)

3 Number of C-terminal heptad-repeats

ND: not determined

A complete sequencing of *futA* and *futB* in five of the subisolates from the persistently infected patient (67:18, 67:19, 67:20, 67:21 and 67:27) revealed genetic modifications that supported the concept that a large number of additional homologous recombination events had occurred between the two genes (Figure S2). In several instances the nucleotide differences were most likely derived through recombination between *futA* or *futB* fragments that were represented by the sequences of the five analyzed isolates. However, in a few cases, it is possible that a DNA fragment was incorporated in the isolates via recombination with an unknown sequence that was not detected in our study. For instance, position 147 to 610 of *futB* in subisolates 67:20 and 67:27 encompassed 14 nucleotide variations that were not shared by any other analyzed subisolates. Likewise, position 240 to 315 of *futA* in 67:18 and 67:19 carried eight unique nucleotide positions. Nevertheless, some of the variation that was represented by isolated base pair differences could also have been derived from point mutation events.

We next performed DNA sequencing of parts of three additional genes (*flaB*, *recA* and *ureI*) that appear once in the *H. pylori* genome without paralogues, and the 16S rRNA gene that is present twice in the *H. pylori* genome and is considered conserved among all isolates of *H. pylori*. From a total of 1879 bp of sequenced DNA only three point mutations in the *ureI* gene could be identified (Table 5). This frequency (0 to 0.51%) was in stark contrast to the *futA*/*futB* situation where modifications were observed at a total of 118 positions (Figure S2), giving frequencies of 8.0% and 4.4% for *futA* and *futB*, respectively (Table 5). When translated to amino acid sequence this corresponded to 43 variable positions at the protein level (Figure S3).

**Table 5:** Genetic changes in the DNA sequence of six *H. pylori* genes from five single-colony isolates.

Gene	Fragment length^1^	No differing nt^2^	Frequency
*futA*	1275	102	8.0%
*futB*	1326	57	4.4%
*flaB*	455	0	0%
*recA*	354	0	0%
*ureI*	586	3	0.51%
16S rRNA	484	0	0%

1 Number of nucleotides sequenced. For *futA* and *futB*, only sequence from the first two heptad-repeats that are common for all isolates were included in the calculations.

2 Number of nucleotides that differed in at least one subisolate.

### Phase variation in FutA and FutB

The expression status of the *futA* and *futB* genes was determined by sequence analysis of the 5′-homopolymeric region, whose number of C-residues alters the reading frame of the gene [40]. The obtained data showed that both 67:20 and 67:21 contained a *futA* that was out-of-frame whereas *futB* was in-frame and thus translated into a full-length protein (Table 1). The same was true for all other isolates from the studied patient, except 67:18, which possessed both *futA* and *futB* in frame. Unexpectedly, this strain did not show any signs of Le^x/y^ expression (Figure 3B). However, this phenotype could be the result of deficiency of, or low activity of, LPS enzymes that act upstream of the FucTs. In fact, 67:18 displayed a lack of high-molecular-weight O-antigen chains (Figure 3A) and this reduction in substrate availability for the FucTs may account for the undetectable levels of Lewis antigens in this specific strain.

Phase variation was also observed in a subset of the isolates derived after *in vitro* passage with a small bottleneck (Table 3). Eight out of the 24 single-cell passaged lineages had an altered number of C-residues in the homopolymeric phase-variable region of either *futA* and/or *futB*. In one isolate (50p 67:21-11), which harbored the corresponding type B allele at both loci, it was likely that homologous recombination between *futA* and *futB*, rather than slipped-strand mispairing, had accounted for the altered number of C-residues in the *futA* gene. Six frame-shifts that were detected in *futB* resulted in a silent copy of the gene and thus no α1,3-FucT activity in the isolates. This corroborated our observations of no Lewis expression in 50pS 67:20-7 and 50pS 67:21-1, -3, -7 and -9. Conversely, we could detect a significant Le^y^-positive O-antigen in 50pS 67:21-8 which, according to the DNA sequencing, possessed both *futA* and *futB* out-of-frame. This finding was surprising since the genotype of the strain suggested it was deprived of any α1,3-FucT activity. One possible explanation for the contradicting results is that, although we only analyzed low passage isolates, the group of bacterial cells that were used for DNA isolation had undergone a phase switch in FutB. This event is possible, but however should occur at low frequency. Evidence of slipped-strand mispairing was also observed in the C-tract of *futA* in two *in vitro* passaged 50pL 67:21 isolates and two *in vivo* mouse-passaged CONV-R 3m isolates (Tables 2 and 4). Nevertheless, none of these alterations resulted in an in-frame variant and thus no change in expression status occurred.

### Altered length of heptad-repeat region in FutA and FutB

We have previously demonstrated that the number of heptad-repeats in FutA and FutB varies within *H. pylori* communities in chronically infected patients *in vivo* [43]. In the present study, the 90-year-old patient was also found to harbor a diverse *H. pylori* population with respect to number of heptad-repeats, as analyzed by PCR and DNA sequencing of the 3′-region of *futA* and *futB*. The most common genotype contained two repeats in *futA* and eight repeats in *futB*. Diversity was observed in four out of twenty isolates and comprised five, fifteen and seventeen repeats in *futA*, while five and seven repeats were detected in the *futB* gene (Table 1). Interestingly, three of these four isolates (67:18, 67:20 and 67:27) showed rearrangements in both genes with an increase in *futA*-repeats and an accompanying decrease in *futB*-repeats.

None of the *in vitro* large bottleneck-derived subisolates revealed any acquired modifications in this region, and were all identical to the corresponding wild-type strains (Table 2). Plasticity was, however, observed in the expressed *futB* gene in seven of the subisolates after 50 *in vitro* passages with a small bottleneck. All isolates with an altered genotype presented a reduction in number of heptads, corresponding to one or two fewer heptads as compared to the *futB* gene in the original isolates of 67:20 and 67:21 (Table 3). Even though non-expressed *futA* of 67:20 carried as many as 17 heptads, this high repeat-number was maintained in all *in vitro* passaged subclones.

Genetic alterations were similarly acquired in the 3′-repetitive region of *futB* in three out of the thirty isolates that were recovered after *in vivo* passage of strain 67:21 in mice. All three isolates (CONV-R 3m-1, -3 and -9) were obtained from the same mouse and carried a modification that corresponded to a reduction in number of heptad-repeats to four heptad-repeats, when compared to the wild-type strain and all additional emerging isolates that expressed an enzyme with eight heptad-repeats (Table 4).

### Correlation between Lewis expression and number of heptad-repeats in FutA and FutB

Diversity in the heptad-repeat region of FutA and FutB affects Lewis glycosylation by a molecular ruler mechanism [43]. This trait was also observed in the subisolates from the current study. The 67:20 and 67:21 isolates carried a silent copy of *futA* and thus only expressed the FutB enzyme, containing five (FutB5) and eight (FutB8) heptad-repeats, respectively (Table 1). The different number of heptad-repeats was reflected in the apparently larger sizes of Le^y^-glycosylated O-antigen chains in isolate 67:21, as compared to the O-antigen chains in isolate 67:20 that were of smaller size (Figure 3B).

Out of the six 50pS 67:20-derived subisolates that significantly expressed Le^x^, two were FutB4 (50pS 67:20-4 and -9) and four were FutB5 (50pS 67:20-5, -10, -11 and -12) (Table 3). This discrepancy of one heptad-repeat was apparent in the immunoblot results where the major Le^x^-expressing O-antigen chain size was one unit shorter in the FutB4 isolates (Figure 5B). Five 50pS 67:21-derived subisolates carried high-molecular-weight Le^y^-glycosylated O-antigen chains. Among these, those that expressed FutB6 and FutB7 (50pS 67:21-6 and -12) displayed a ladder pattern with the most prominent Le^y^-glycosylated bands of smaller molecular weight than the corresponding isolates with FutB8 (50pS 67:21-2, -4 and -10) (Figure 5B). The three 67:21-derived isolates that lost *futB*-repeats during *in vivo* passage in mice (CONV-R 3m-1, -3 and -9) all expressed FutB4 (Table 4). Phenotypically, these FutB4 isolates carried an LPS molecule with Le^y^-glycosylated O-antigen polymers of lower molecular weight than the remaining Le^y^-positive FutB8 isolates (Figure 6B). Moreover, such correlation between number of heptad-repeats and sizes of O-antigen chains was also observed in silver staining of total LPS, where the sizes of the dominant high-molecular-weight O-antigen chains corresponded to the number of heptad-repeats (Figure S1).

## Discussion

Most clinical *H. pylori* isolates express Lewis antigens on their LPS molecules [21], [32], [48]. However, considerable phenotypic variations exist between Lewis-expressing isolates, both in the specificity of saccharides that are used as building blocks, as well as in the number and order of these units. Such diversity is most prominent between unrelated strains, but also occurs within defined *H. pylori* communities of highly related subisolates [11], [43]–[45], [49], [50]. Here, we corroborate the presence of bacterial micro-diversity and show that phenotypically distinct LPS molecules arise and are maintained within *H. pylori* populations in the human gastric mucosa, and are also generated after *in vitro* subculturing and *in vivo* transfer to a new animal host.

Isolates of *H. pylori* express either rough- or smooth- LPS and the vast majority of clinical isolates belong to the latter group with expression of an extended O-antigen chain [22], [32]. Analyses of clinical *H. pylori* isolates have demonstrated that cells that have undergone phase-variation and no longer express Lewis antigens still produce an elongated O-antigen chain [43], [50], [51]. Thus, it could be deduced that loss of O-antigen chain expression is not common among individual cells in *H. pylori* communities in the human gastric mucosa, and most isolates express an elongated O-antigen chain. However, *in vitro* growth conditions have been proposed to reversibly affect the production of the O-antigen chain. Smooth strains that are repeatedly subcultured on solid media may lose their ability to produce O-antigen chains, and this rough phenotype can be reversed by a single passage in liquid media [22], [24]. We subcultured two clinical isolates 50 times on agar medium with either a large (50pL) or small (50pS) bottleneck. Interestingly, after propagation with a large bottleneck, the bacteria retained the smooth O-antigen chain-expressing phenotype, even without a final passage in broth, and no inverse selection against O-antigen chain expression had occurred.

Nevertheless, major changes in the O-antigen chain were observed after passage of strain 67:21 *in vitro* with a small bottleneck, as well as *in vivo* in mice. Eight of the 67:21 50pS isolates, and all but two of the mouse-passaged isolates, differed in the O-antigen chain ladder pattern, and either lacked or demonstrated relatively low expression of several prominent high-molecular-weight O-antigen chain bands, respectively. These variants may have appeared spontaneously through mutations or phase variation and it should not be ruled out that the occurrence of such phenotypes after *in vitro* and *in vivo* passage could be due to overgrowth of certain genotypes at random. However, a more likely explanation might be that there is a selection for expression of smooth-LPS carrying high-molecular-weight O-antigen chains *in vivo* in the human gastric mucosa, and that this selective pressure is not present *in vitro* or *in vivo* in non-human mammalian gastric environments. The O-antigen chain, thus, may be important for *H. pylori* survival and persistence in the human stomach, but outside this environment, where there is no advantage of O-antigen chain expression, the bacterium will lose this property. Since this reaction is reversible *in vitro* [24] it can also be assumed that a rough or semi-rough isolate will switch to smooth-LPS expression once it encounters the gastric mucosa upon transmission to a new human host. The genetic basis for this switch is currently unknown.

By comparing the levels of acquired diversity after the three different modes of subculturing, it is notable that the phenotypes were most stable after *in vitro* passages using a large bottleneck, as compared to *in vitro* passage with a small bottleneck or *in vivo* passage in mice. The difference between the two approaches of *in vitro* subculturing is the number of bacteria that are being transferred in each step. Subculturing with a small bottleneck will purify and select for variants that would disappear in a larger population, which could explain why we observed more diversity after passages of single-cell colonies as compared to sweeps.

In the case of *in vivo* passage in mice, the infecting strain was a single-cell colony isolate with limited preceding subculturing steps. Thus, the variants were most likely not present at the time of infection, but evolved during the course of colonization in the murine stomach. We do not known whether these variants arose shortly after infection onset, due to a rapid adaptation to the environment in the new host, or if they evolved over a prolonged period of time. It is, however, likely that the swift change in surrounding conditions after transfer to another host selects for variants with characteristics that are better accustomed to the new milieu. It has been proposed that *H. pylori* adapts to the host by changing its Lewis expression to match that of the colonized gastric mucosa [52], [53]. This mechanism of molecular mimicry could be a means for the bacterium to evade immune recognition by the host, but contradicting data has been presented [31], [54] and it is thus uncertain if such adaptation occurs in a human infection. In our experimental animal infection, the mice expressed Le^b^ in the gastric mucosa and a molecular mimicry effect would thus select for Le^y^-expressing *H. pylori* isolates. However, a reduction in Le^y^-expression was observed in the *H. pylori* isolates after transfer to the murine stomach, arguing against Lewis molecular mimicry in this case. Each of the three mice included in this study were unique with respect to time of infection, and presence or absence of a normal microflora, but the small number of mice did not allow us to draw any firm conclusions about the effect of these conditions.

Development of gastric pathology in an infected human is significantly associated with the presence of the *cag* PAI in the colonizing *H. pylori* isolates [5]. A correlation with Lewis antigen expression in such isolates has also been suggested. This concept was supported by a positive relationship between *cagA* status and Lewis phenotype in clinical *H. pylori* isolates, as well as in a wildtype and isogenic *cagA* knockout mutant pair [27], [46] Nevertheless, this correlation is not always obvious, but appears to vary between human populations [46], [55]. Here, we analyzed 20 single-colony isolates from one individual, of which half were *cag* PAI positive. All but one of these isolates expressed Le^y^ in various amounts, but no correlation to *cagA* status could be discerned. This data was obtained from only one infected patient and it can not be excluded that a correlation might be recognized in samples obtained from an extended number of individuals. However, the observations from these subisolates, all with indistinguishable genomic fingerprints, argue against a difference in Le^y^-expression in naturally occurring *cagA* subvariants.

*H. pylori* possesses two α1,3/4-FucTs, FutA and FutB. Though they at a first glance appear to carry out seemingly identical functions, the enzymes often differ in activity and specificity [32], [34], [36], [56] and the dual components may be required to create diversity in O-antigen chains. The two genes are homologous, and sequence diversity is often no more pronounced between the *futA* and *futB* genes within an isolate than between the corresponding genes of unrelated strains [36], [56]. Thus, recombination events could be predicted to occur both within a genome as well as between different isolates after transformation of foreign DNA. In this study, we observed subvariants carrying chimeric *futA* and *futB* that co-existed within an *H. pylori* population in a human stomach. These chimeras presumably arose from homologous recombination between *futA* and *futB*, and we report evidence for such events occurring both *in vivo* and *in vitro*. In a few instances, it was likely that recombination with an unknown fragment, which was not detected by sequencing in our study, had occurred. Such fragments could have been present in *H. pylori* cells that colonized or transiently occupied the stomach in the past. It has been shown that *H. pylori* uses exceptionally short DNA fragments for homologous recombination events [13], [57]. The observations from our study support this concept, as multiple recombination events between *futA* and *futB* yielded exchange of rather short gene segments. In addition, mutations could also occasionally account for the detected diversity in *futA* and *futB* [20]. Depending on the quality and quantity of alterations in the amino acid sequence, exchange of partly homologous DNA and mutations could generate minor or major changes in the FucT activity of the isolates, and thus affect the presentation of surface molecules to the bacterial environment.

The observed genetic rearrangements in *futA* and *futB* of the studied isolates were remarkably high. In order to reveal if this was a common feature throughout the genomes of these subisolates, or an attribute restricted to these loci, we analyzed sequences from four additional genetic regions. The relatively low frequency of genetic modifications outside the *futA* and *futB* loci supported our hypothesis of homologous recombination events taking place at a high frequency between the two paralogous α1,3-FucTs.

We have previously described how the number of C-terminal heptad-repeats in FutA and FutB varies within and between strains in chronically infected patients [43], and such diversity was further supported by analysis of the *H. pylori* communities in the present investigation. Different scenarios might predispose for rearrangement in this region and the repeat units might undergo duplication or deletion through (i) slipped-strand mispairing events within a gene during recombination, (ii) intra-genomic homologous recombination between *futA* and *futB* within a single isolate or (iii) inter-genomic homologous recombination of either *futA* or *futB* fragments after transformation of genomic DNA from a foreign isolate. Within the *H. pylori* population that persistently colonized the patient from the present study, most isolates shared a common genotype, with respect to number of heptad-repeats, but four out of twenty isolates differed in this manner. An interesting observation is that, in three out of these four isolates, rearrangements were revealed concurrently in *futA* and *futB*. This implies that there was a link between the two paralogous genes in the generation of such diversity. It is thus likely that, in these cases, the change in number of heptad-repeats occurred simultaneously through homologous recombination between the *futA* and *futB* loci within one isolate. However, after *in vitro* and *in vivo* passages, such changes were only observed in one of the two genes, implicating another mechanism, and repeat units were possibly deleted through intragenic slippage during replication.

The amount of heptad-repeats in FutA and FutB have previously been described to range from one to ten in various *H. pylori* isolates [36], [43]. Notably, two of the clinical isolates that were analyzed in the present study were found to possess as many as 15 and 17 repeats in *futA*. A DNA sequence that possesses a large number of repetitive elements would theoretically be more prone to internal rearrangement events than a corresponding sequence with fewer repeats. Nevertheless, in the case of 67:20, the sequence of *futA* that carried 17 repeats was stable during the course of 50 steps of *in vitro* subculturing. Moreover, it is noteworthy that, subsequent to both *in vitro* and *in vivo* passages, all genetic alterations that were observed in this region occurred in the *futB* gene, which was the single in-frame α1,3-FucT of both 67:20 and 67:21. Consequently, there might be an advantage and a selection for changing the number of heptad-repeats in expressed α1,3-FucTs, whereas silent copies remain unaffected. Alternatively, phase variation in *futA* and *futB* could indirectly influence recombination events within the gene. In-frame variants, such as *futB* in this case, would exhibit higher levels of transcription, which might stimulate both recombination and mutation rates within that particular gene [58]. It remains to be verified whether this is the case within the *futA* and *futB* genes of *H. pylori*.

The C-terminus of the *H. pylori* α1,3-FucTs has been shown to be important for stability of the protein, and both secondary and quaternary structure require the presence of at least part of the heptad-repeats [59]. Consequently, removal of all heptad-repeats lead to major structural alterations and corresponding loss of enzymatic activity [59], [60]. Moreover, the heptad-repeats have been suggested to form a dimerization domain [34], [59] and such a role has also been supported by experimental *in vitro* data [59]. We have previously presented a model in which the heptad-repeat region constitutes a domain that can be involved in forming either homo- and/or hetero-dimers, depending on the number of heptad-repeats and expression status of the two α1,3-FucT enzymes [43]. Data from the current study corroborates the interpretation of this region as a molecular ruler, with an observed correlation between Lewis glycosylation pattern and number of heptad-repeats in subcultured isolates. This model is based on *in vivo* observations in *H. pylori* cells, and further biochemical analyses would be required to further elucidate the role of the heptad-repeat domain. One limitation in the studies of *H. pylori* FucTs is that so far it has not been possible to express and purify *in vitro* full-length enzymes. Thus, either C-terminal truncated proteins or bacterial lysates have been used for structural and enzymatic studies [36], [42], [56], [61]. Data derived thereof give important information about the function and activity of the enzymes, but might also differ from the *in vivo* situation when the enzymes are active in their natural environment, i.e. the intact bacterial membrane. Therefore, *in vivo* observations are invaluable and should function to complement biochemical studies.

It is still not fully recognized how *H. pylori* successfully colonizes more than half the human population. This bacterium is able to survive attempts of eradication from the host immune system, and causes a persistent infection that follows an individual for life. Many factors likely contribute to colonization and subsequent disease development. Host genetics and environmental factors play important roles, but the substantive genotypic and phenotypic variation between *H. pylori* isolates presumably facilitates colonization in the human gastric mucosa [62]–[64]. Although this is a distinct niche, the environment differs between individuals as well as within a person over the years of aging [4], [64]. Thus, when the bacterium is transmitted between individuals, a new set of characteristics may be required to establish infection in the new host. Moreover, in parallel with developmental and environmental changes in the stomach, the bacterium has to adapt to the new conditions. A constantly evolving population of bacteria creates a mixture of cells with micro-diversity in their genetic setup. Individual cells are better or worse fit for environmental changes, but the likelihood of the bacterial community *per se* to survive is immensely increased by intra-population diversity. Thus, a certain level of diversity ought to be sustained in order to retain the ability of the population to swiftly adapt to changes in the environment.

LPS is a surface structure and as such possesses the ability to interact with host cells or become recognized by the host immune system [21], [32], [65], [66]. Lewis antigen phase variation [40], [41], [44] and display of structural LPS variants [32] may thus provide a means to regulate the inflammatory response provoked in the host. It is conceivable that it is beneficial for the bacterium to control the level of inflammation and that the optimal balance changes over the course and duration of infection. Hence, diversification in the LPS molecule might be crucial both in the initial colonization of a new niche as well as the establishment of persistent infection.

In conclusion, in this study we have described *H. pylori* LPS diversity in a set of related subclones generated *in vivo* in a persistent human infection, as well as after experimental *in vitro* and *in vivo* passages. The character and frequency of diversification may depend on the transfer bottleneck and environmental conditions. Moreover, genetic rearrangements, such as phase variation and recombination events, in the two α1,3-FucTs account for a substantial level of the created LPS diversity. The surface localization of LPS O-antigen chains displays an interaction domain to the surrounding milieu, and its variability may be fundamental for the bacterium to adapt to changing environments within the gastric mucosa.

## Materials and Methods

### *H. pylori* strains and culture conditions

*H. pylori* cells were cultured from gastric biopsies obtained from a 90-year old female patient who was suffering from gastric ulcer. This patient was recruited to a Swedish cancer case-control study that was approved by the ethics committee of the Medical Faculty, Uppsala University (Uppsala, Sweden) and has been described elsewhere [15], [67]. Single-cell colonies of *H. pylori* were isolated from the primary culture and grown on agar medium under microaerophilic conditions [15]. The number of *in vitro* passages was kept to a minimum unless otherwise stated. Prior to LPS isolation, the bacteria were cultured in *Brucella* broth in order to enrich for O-antigen chain expression [43]. Extensive genetic characterization and mouse colonization studies of subclones 67:20 and 67:21 have been described elsewhere [15]. For investigation of IL-8 induction, *H. pylori* isolates were co-cultured for 6 h with the human gastric epithelial cell line AGS, whereafter levels of IL-8 in the media were measured [68].

### *In vitro* and *in vivo* passage of isolates 67:20 and 67:21

The *H. pylori* isolates 67:20 and 67:21 were subcultured by 50 *in vitro* passages on agar medium using two approaches. First, a sweep of bacteria was transferred in each passage and twelve individual single colonies were isolated after the final passage. Second, twelve single primary colonies were isolated from 67:20 and 67:21, and subcultured in parallel for 50 passages, transferring one single colony in each step. For both approaches, the bacteria were passaged every 3–4 days to sustain fresh cultures of *H. pylori*. Furthermore, isolate 67:21 was used for *in vivo* passage in transgenic mice, expressing the Le^b^ receptor on gastric pit and surface mucous cells [15], [47]. Details of the mouse infection experiments have previously been published [15], [47]. For the present study, three mice were selected, one ex-germ-free mouse, mono-associated with *H. pylori* 67:21 for three months (GF 3m), and two conventionally raised mice, infected with 67:21 for three (CONV-R 3m) and ten months (CONV-R 10m), respectively. Ten single-colony re-isolates of 67:21 were recovered from each mouse for the subsequent analyses. All animal experiments were approved by the local animal ethics committee, Karolinska Institutet, Stockholm, Sweden.

### Isolation and phenotypic analyses of *H. pylori* LPS

LPS was isolated from the *H. pylori* isolates, using hot phenol/water extraction, as previously described [43]. The purified LPS preparations were separated by SDS polyacrylamide gel electrophoresis (SDS-PAGE) using a 4% stacking layer and a 15% separation gel. Gels were stained with silver (BioRad, Hercules, CA) or blotted to PVDF membrane (BioRad) for immunodetection. Primary antibodies used were mouse monoclonal anti-CD15 (i.e. Le^x^) or anti-Le^y^ (both from Serotec, Oxford, UK), and secondary antibody was rabbit anti-mouse, conjugated to horseradish peroxide (DAKO, Glostrup, Denmark). Enzyme-linked immunosorbent assay (ELISA) was performed as previously described [55] on a subset of isolates to confirm the results from the immunoblot experiments (isolates 67:20, 67:21, 50pS 67:21-1, -2, -3, -4, -5 and -6, CONV-R 3m-2, -3, -4, -5 and -6, and GF 3m-7, -8 and -9). The antibodies used were IgM mouse monolclonal antibodies against Le^a^ (clone T174), Le^b^ (clone T128), Le^x^ (clone P12), Le^y^ (clone F3) and sialyl-Le^x^ (clone CSLEX1) from Singnet laboratories (Dedhan, MA). The two methods showed good agreement in the detection of Le^y^ in the isolates. In a one case (isolate GF 3m-8), Le^x^ was detected by ELISA but not by immunoblot, which could reflect differences in sensitivity between the two methods. None of the analyzed isolates were positive for Le^a^, Le^b^ or sialyl-Le^x^.

### Genetic analyses

Genomic DNA was isolated with a DNeasy® Tissue Kit (Qiagen, Hilden, Germany), using the protocol for Gram-negative bacteria. DNA fingerprints were analyzed by arbitrary primed PCR using the PMAV17B primer, as previously described [10]. Primers that were used for either PCR and/or DNA sequencing were designed using the Primer3 software and are listed in Table S1. PCR amplifications were performed under standard conditions with DyNAzyme Taq polymerase and buffer (Finnzymes, Espoo, Finland). Cycle sequencing reactions were carried out using BigDye® Terminator v3.1 Cycle Sequencing Kit (Applied Biosystems, Foster City, CA) and sequencing analyses were performed on ABI PRISM® 3100 Genetic Analyzer (Applied Biosystems). All DNA sequences were assembled and aligned using the Vector NTI 10.3.0 software package.

## Supporting Information

Figure S1 Comparison of LPS profiles from various samples included in Figures 3–6. For comparisons of different sets of isolates between experiments, selected LPS samples were analyses in parallel in the same gel and stained with silver. Number of heptad-repeats in the expressed FutB enzyme is shown below the lanes.
(3.28 MB EPS)

Figure S2 DNA sequence of the *futA* and *futB* genes from five single-colony isolates. The complete *futA* and *futB* genes were sequenced in subisolates 67:18, 67:19, 67:20, 67:21 and 67:27. A total of 118 nucleotide positions were found to differ in at least one isolate. This calculation excludes the sequence between positions 1138–1452 that encode heptad-repeats that are not shared by all the isolates, i.e. only the first two repeat units that were common for all strains are included.
(0.23 MB PDF)

Figure S3 Amino acid sequence of FutA and FutB in five single-colony isolates. The DNA sequence from subisolates 67:18, 67:19, 67:20, 67:21 and 67:27 were translated into amino acid sequence. For comparisons of in-frame sequences, one C-residue was manually inserted or deleted in the C-tract of futA sequences of 67:19, 67:20, 67:21 and 67:27 before translation into protein sequence. A total of 43 amino acid positions were found to differ in at least one isolate. This calculation excludes the heptad-repeat sequence between positions 380–484 that is not shared by all isolates.
(0.10 MB PDF)

Table S1 Primers used in this study
(0.05 MB PDF)
