# Supplementary material for: Lipopolysaccharide Diversity Evolving in Helicobacter pylori Communities through Genetic Modifications in Fucosyltransferases
Source: PLoS One. 2008 Nov 26;3(11):e3811. doi: 10.1371/journal.pone.0003811 (PMC2583950; doi:10.1371/journal.pone.0003811)
Supplement: Figure S2 — DNA sequence of the futA and futB genes from five single-colony isolates. The complete futA and futB genes were sequenced in subisolates 67:18, 67:19, 67:20, 67:21 and 67:27. A total of 118 nucleotide positions were found to differ in at least one isolate. This calculation excludes the sequence between positions 1138–1452 that encode heptad-repeats that are not shared by all the isolates, i.e. only the first two repeat units that were common for all strains are included. (0.23 MB PDF) [file pone.0003811.s002.pdf]

```
                 (1) 1         10        20        30        40        50        60        70        80        90       100
futA 67:18       (1) ATGTTCCAACCCTTACTAGACGCCTTTATAGAAAGCGCTCCAATTAAAAAAAAATTACCTTTAAATCTCCCCCCCCCCCCCCTAAAAATCGCTGTGGCGA
futA 67:19       (1) ATGTTCCAACCCTTACTAGACGCCTTTATAGAAAGCGCTCCAATTAAAAAAAAATTACCTTTAAATCTCCCCCCCCCCCCC-TAAAAATCGCTGTGGCGA
futA 67:20       (1) ATGTTCCAACCCTTACTAGACGCCTTCATAGAAAGCGCTTCCATTGAAAAA--ATGGCCTCTAAATCTCCCCCCCCCCCC--TAAAAATCGCTGTGGCGA
futA 67:21       (1) ATGTTCCAACCCTTACTAGACGCCTTTATAGAAAGCGCTCCAATTAAAAAAAAATTACCTTTAAATCTCCCCCCCCCCCC--TAAAAATCGCTGTGGCGA
futA 67:27       (1) ATGTTCCAACCCTTACTAGACGCCTTCATAGAAAGCGCTTCCATTGAAAAA--ATGGCCTCTAAATCTCCCCCCCCCCCC--TAAAAATCGCTGTGGCGA
futB 67:18       (1) ATGTTCCAACCCTTACTAGACGCCTTCATAGAAAGCGCTTCCATTGAAAAA--ATGGCCTCTAAATCTCCCCCCCCCCCCC-TAAAAATCGCTGTGGCGA
futB 67:19       (1) ATGTTCCAACCCTTACTAGACGCCTTCATAGAAAGCGCTTCCATTGAAAAA--ATGGCCTCTAAATCTCCCCCCCCCCCCC-TAAAAATCGCTGTGGCGA
futB 67:20       (1) ATGTTCCAACCCTTACTAGACGCCTTCATAGAAAGCGCTTCCATTGAAAAA--ATGGCCTCTAAATCTCCCCCCCCCCCCC-TAAAAATCGCTGTGGCGA
futB 67:21       (1) ATGTTCCAACCCTTACTAGACGCCTTCATAGAAAGCGCTTCCATTGAAAAA--ATGGCCTCTAAATCTCCCCCCCCCCCCC-TAAAAATCGCTGTGGCGA
futB 67:27       (1) ATGTTCCAACCCTTACTAGACGCCTTCATAGAAAGCGCTTCCATTGAAAAA--ATGGCCTCTAAATCTCCCCCCCCCCCCC-TAAAAATCGCTGTGGCGA

               (101) 101       110       120       130       140       150       160       170       180       190      200
futA 67:18     (101) ATTGGTGGGGAGGC------GCTGAAGAATTTAAAAAGAGCACTTTATATTTCATCCTAAGCCAACGCTACACAATCACCCTCCACCAAAACCCCAACAA
futA 67:19     (100) ATTGGTGGGGAGGC------GCTGAAGAATTTAAAAAGAGCACTTTATATTTCATCCTAAGCCAACGCTACACAATCACCCTCCACCAAAACCCCAACAA
futA 67:20      (97) ATTGGTGGAGAGATAAAGAGGTTAAAGAATTTAAAAAGAGCGTTCTTTATTTTATCCTAAGCCAACGCTACACAATCACCCTCCACCAAAACCCCAACAA
futA 67:21      (99) ATTGGTGGAGAGGC------GCTGAAGAATTTAAAAAGAGCACTTTATATTTCATCCTAAGCCAACGCTACACAATCACCCTCCACCAAAACCCCAACGA
futA 67:27      (97) ATTGGTGGAGAGATAAAGAGGTTAAAGAATTTAAAAAGAGCGTTCTTTATTTTATCCTAAGCCAACGCTACACAATCACCCTCCACCAAAACCCCAACAA
futB 67:18      (98) ATTGGTGGAGAGATAAAGAGGTTAAAGAATTTAAAAAGAGCGTTCTTTATTTTATCCTAAGCCAACGCTACACAATCACCCTCCACCAAAACCCCAACAA
futB 67:19      (98) ATTGGTGGAGAGATAAAGAGGTTAAAGAATTTAAAAAGAGCGTTCTTTATTTTATCCTAAGCCAACGCTACACAATCACCCTCCACCAAAACCCCAACAA
futB 67:20      (98) ATTGGTGGGGAGGC------GCTGAAGAATTTAAAAAGAGCACTCTGTATTTTATCTTAAGCCAACGCTACACAATCACCCTCCACCAAAACCCCAACAA
futB 67:21      (98) ATTGGTGGAGAGATAAAGAGGTTAAAGAATTTAAAAAGAGCGTTCTTTATTTTATCCTAAGCCAACGCTACACAATCACCCTCCACCAAAACCCCAACAA
futB 67:27      (98) ATTGGTGGGGAGGC------GCTGAAGAATTTAAAAAGAGCACTCTGTATTTTATCTTAAGCCAACGCTACACAATCACCCTCCACCAAAACCCCAACAA

               (201) 201       210       220       230       240       250       260       270       280       290      300
futA 67:18     (195) ACCCTCCGATCTCGTCTTTGGCAGTCCTATTGGATCAGCCAGAAAAATCCTATCCTATCAAAACACTAAACGAGTGTTTCACACAGGTGAAAATGAAGTC
futA 67:19     (194) ACCCTCCGATCTCGTCTTTGGCAGTCCTATTGGATCAGCCAGAAAAATCCTATCCTATCAAAACACTAAACGAGTGTTTCACACAGGTGAAAATGAAGTC
futA 67:20     (197) ACCCTCCGATCTCGTCTTTGGCAATCCTATTGGAGCAGCTAGAAAAATCTTATCCTATCAAAACACTAAAAGGGTGTTTTACACCGGTGAGAACGAAGTC
futA 67:21     (193) ACCCTCCGATCTCGTCTTTGGCAGTCCTATTGGATCAGCTAGAAAAATCTTATCCTATCAAAACACTAAAAGGGTGTTTTACACCGGTGAGAACGAAGTC
futA 67:27     (197) ACCCTCCGATCTCGTCTTTGGCAATCCTATTGGAGCAGCTAGAAAAATCTTATCCTATCAAAACACTAAAAGGGTGTTTTACACCGGTGAGAACGAAGTC
futB 67:18     (198) ACCCTCCGATCTCGTCTTTGGCAATCCTATTGGAGCAGCTAGAAAAATCTTATCCTATCAAAACACTAAAAGGGTGTTTTACACCGGTGAGAACGAAGTC
futB 67:19     (198) ACCCTCCGATCTCGTCTTTGGCAATCCTATTGGAGCAGCTAGAAAAATCTTATCCTATCAAAACACTAAAAGGGTGTTTTACACCGGTGAGAACGAAGTC
futB 67:20     (192) ACCCTCCGATCTCGTCTTTGGCAATCCTATTGGAGCAGCTAGAAAAATCTTATCCTATCAAAACACTAAAAGGGTGTTTTACACCGGCGAAAACGAAGTC
futB 67:21     (198) ACCCTCCGATCTCGTCTTTGGCAATCCTATTGGAGCAGCTAGAAAAATCTTATCCTATCAAAACACTAAAAGGGTGTTTTACACCGGTGAGAACGAAGTC
futB 67:27     (192) ACCCTCCGATCTCGTCTTTGGCAATCCTATTGGAGCAGCTAGAAAAATCTTATCCTATCAAAACACTAAAAGGGTGTTTTACACCGGCGAAAACGAAGTC

               (301) 301       310       320       330       340       350       360       370       380       390      400
futA 67:18     (295) CCTAATTTCAACCTTTTTGATTACGCCATAGGCTTTGATGAATTGGACTTTAGAGATCGTTATTTGAGAATGCCTTTATATTATGATAGACTACACCATA
futA 67:19     (294) CCTAATTTCAACCTTTTTGATTACGCCATAGGCTTTGATGAATTGGACTTTAGAGATCGTTATTTGAGAATGCCTTTATATTATGATAGACTACACCATA
futA 67:20     (297) CCTAATTTCAACCTCTTTGATTACGCCATAGGCTTTGATGAATTGGATTTTAGAGATCGTTATTTGAGAATGCCTTTATATTATGATAGACTACACCATA
futA 67:21     (293) CCTAATTTCAACCTCTTTGATTACGCCATAGGCTTTGATGAATTGGATTTTAGAGATCGTTATTTGAGAATGCCTTTATATTATGATAGACTACACCATA
futA 67:27     (297) CCTAATTTCAACCTCTTTGATTACGCCATAGGCTTTGATGAATTGGATTTTAGAGATCGTTATTTGAGAATGCCTTTATATTATGATAGACTACACCATA
futB 67:18     (298) CCTAATTTCAACCTCTTTGATTACGCCATAGGCTTTGATGAATTGGATTTTAGAGATCGTTATTTGAGAATGCCTTTATATTATGATAGACTACACCATA
futB 67:19     (298) CCTAATTTCAACCTCTTTGATTACGCCATAGGCTTTGATGAATTGGACTTTAGAGATCGTTATTTGAGAATGCCTTTATATTATGATAGACTACACCATA
futB 67:20     (292) CCTAACTTCAACCTCTTTGATTACGCCATAGGCTTTGATGAATTAGACTTTAGAGATCGTTATTTGAGAATGCCTTTATATTATGATAGACTACACCATA
futB 67:21     (298) CCTAATTTCAACCTCTTTGATTACGCCATAGGCTTTGATGAATTGGATTTTAGAGATCGTTATTTGAGAATGCCTTTATATTATGATAGACTACACCATA
futB 67:27     (292) CCTAACTTCAACCTCTTTGATTACGCCATAGGCTTTGATGAATTAGACTTTAGAGATCGTTATTTGAGAATGCCTTTATATTATGATAGACTACACCATA

               (401) 401       410       420       430       440       450       460       470       480       490      500
futA 67:18     (395) AAGCAGAGAGCGTGAATGACACCACCGCACCCTACAAGATTAAAGATAACAGCCTTTATACTTTAAAAAAACCCACCCATTGTTTTAAAGAAAACCACCC
futA 67:19     (394) AAGCCGAGAGCGTGAATGACACCACTGCGCCCTACAAGATTAAAGACAACAGCCTTTATACTTTAAAAAAACCCTCCCATTGTTTTAAAGAAAACCACCC
futA 67:20     (397) AAGCAGAGAGCGTGAATGACACCACCGCACCCTACAAGATTAAAGATAACAGCCTTTATACTTTAAAAAAACCCACCCATTGTTTTAAAGAAAACCACCC
futA 67:21     (393) AAGCCGAGAGCGTGAATGACACCACTGCGCCCTACAAGATTAAAGACAACAGCCTTTATACTTTAAAAAAACCCTCCCATCATTTTAAAGAAAACCACCC
futA 67:27     (397) AAGCAGAGAGCGTGAATGACACCACCGCACCCTACAAGATTAAAGATAACAGCCTTTATACTTTAAAAAAACCCACCCATTGTTTTAAAGAAAACCACCC
futB 67:18     (398) AAGCAGAGAGCGTGAATGACACCACCGCACCCTACAAGATTAAAGATAACAGCCTTTATACTTTAAAAAAACCCACCCATTGTTTTAAAGAAAACCACCC
futB 67:19     (398) AAGCCGAGAGCGTGAATGACACCACTGCGCCCTACAAGATTAAAGACAACAGCCTTTATACTTTAAAAAAACCCTCCCATTGTTTTAAAGAAAACCACCC
futB 67:20     (392) AAGCCGAGAGCGTGAATGACACCACCGCACCCTACAAGATCAAATCTGACAGCCTTTATGCTTTAAAAAAACCCTCCCATCATTTTAAAGAAAACCACCC
futB 67:21     (398) AAGCAGAGAGCGTGAATGACACCACCGCACCCTACAAGATTAAAGATAACAGCCTTTATACTTTAAAAAAACCCACCCATTGTTTTAAAGAAAACCACCC
futB 67:27     (392) AAGCCGAGAGCGTGAATGACACCACCGCACCCTACAAGATCAAATCTGACAGCCTTTATGCTTTAAAAAAACCCTCCCATCATTTTAAAGAAAACCACCC

               (501) 501       510       520       530       540       550       560       570       580       590      600
futA 67:18     (495) CCATTTATGCGCAGTAGTGAATAATGAGAGCGATCCTTTGAAAAGAGGGTTTGCCAGTTTTGTCGCTAGCAACCCTAACGCCCCTAAAAGAAACGCTTTC
futA 67:19     (494) CCATTTATGCGCAGTAGTGAATAATGAGAGCGATCCTTTGAAAAGAGGGTTTGCCAGTTTTGTCGCTAGCAACCCTAACGCCCCTAAAAGAAACGCTTTC
futA 67:20     (497) CCATTTATGCGCAGTAGTGAATAATGAGAGCGATCCTTTGAAAAGAGGGTTTGCCAGTTTTGTCGCTAGCAACCCTAACGCCCCTAAAAGAAACGCTTTC
futA 67:21     (493) CCATTTATGTGCAGTAGTGAATAACGAGAGCGATCCTTTGAAAAGAGGGTTTGCGAGTTTTGTCGCGAGCAACGCTAACGCTCCTATGAGGAACGCTTTC
futA 67:27     (497) CCATTTATGCGCAGTAGTGAATAATGAGAGCGATCCTTTGAAAAGAGGGTTTGCCAGTTTTGTCGCTAGCAACCCTAACGCCCCTAAAAGAAACGCTTTC
futB 67:18     (498) CCATTTATGCGCAGTAGTGAATAATGAGAGCGATCCTTTGAAAAGAGGGTTTGCCAGTTTTGTCGCTAGCAACCCTAACGCCCCTAAAAGAAACGCTTTC
futB 67:19     (498) CCATTTATGCGCAGTAGTGAATAATGAGAGCGATCCTTTGAAAAGAGGGTTTGCCAGTTTTGTCGCTAGCAACCCTAACGCCCCTAAAAGAAACGCTTTC
futB 67:20     (492) CCATTTATGCGCAGTAGTGAATGATGAGAGCGATCCTTTGAAAAGAGGGTTTGCCAGTTTTGTCGCAAGCAATCCTAACGCTCCTAAAAGGAACGCTTTC
futB 67:21     (498) CCATTTATGCGCAGTAGTGAATAATGAGAGCGATCCTTTGAAAAGAGGGTTTGCCAGTTTTGTCGCTAGCAACCCTAACGCCCCTAAAAGAAACGCTTTC
futB 67:27     (492) CCATTTATGCGCAGTAGTGAATGATGAGAGCGATCCTTTGAAAAGAGGGTTTGCCAGTTTTGTCGCAAGCAATCCTAACGCTCCTAAAAGGAACGCTTTC
```

```
(601)           601       610       620       630       640       650       660       670       680       690      700
futA 67:18 (595) TATGAGGCTTTAAACGCTATTGAGCCAGTTACTGGGGGAGGGAGCGTGAAAAACACTTTAGGCTATAATGTCAAAAACAAGAGCGAGTTTTTAAGCCAAT
futA 67:19 (594) TATGACGCTTTAAATTCTATTGAGCCGGTTGCTGGGGGAGGAGCAGTAAAAAACACTTTAGGCTATAACGTCAAAAACAAGAGCGAGTTTTTAAGCCAAT
futA 67:20 (597) TATGACGCTTTAAATTCTATTGAGCCGGTTGCTGGGGGAGGAGCAGTAAAAAACACTTTAGGCTATAACGTCAAAAACAAGAGCGAGTTTTTAAGCCAAT
futA 67:21 (593) TATGAGGCTTTAAACGCTATTGAGCCAGTTACTGGGGGAGGGAGCGTGAAAAACACTTTAGGCTATAATGTCAAAAACAAGAGCGAGTTTTTAAGCCAAT
futA 67:27 (597) TATGACGCTTTAAATTCTATTGAGCCGGTTGCTGGGGGAGGAGCAGTAAAAAACACTTTAGGCTATAACGTCAAAAACAAGAGCGAGTTTTTAAGCCAAT
futB 67:18 (598) TATGAGGCTTTAAACGCTATTGAGCCAGTTACTGGGGGAGGGAGCGTGAAAAACACTTTAGGCTATAATGTCAAAAACAAGAGCGAGTTTTTAAGCCAAT
futB 67:19 (598) TATGACGCTTTAAATTCTATTGAGCCGGTTGCTGGGGGAGGAGCAGTAAAAAACACTTTAGGCTATAACGTCAAAAACAAGAGCGAGTTTTTAAGCCAAT
futB 67:20 (592) TATGACGCTCTAAATTCTATTGAGCCGGTTGCTGGGGGAGGAGCAGTGAAAAACACTTTAGGCTATAATGTCAAAAACAAGAGCGAGTTTTTAAGCCAAT
futB 67:21 (598) TATGAGGCTTTAAACGCTATTGAGCCAGTTACTGGGGGAGGGAGCGTGAAAAACACTTTAGGCTATAATGTCAAAAACAAGAGCGAGTTTTTAAGCCAAT
futB 67:27 (592) TATGACGCTCTAAATTCTATTGAGCCGGTTGCTGGGGGAGGAGCAGTGAAAAACACTTTAGGCTATAATGTCAAAAACAAGAGCGAGTTTTTAAGCCAAT

(701)           701       710       720       730       740       750       760       770       780       790      800
futA 67:18 (695) ACAAATTCAATCTGTGTTTTGAAAACTCGCAAGGCTATGGCTATGTAACTGAAAAAATCATTGACGCTTATTTCAGCCATACCATTCCCATTTATTGGGG
futA 67:19 (694) ACAAATTCAATCTGTGTTTTGAAAACACTCAAGGCTATGGCTATGTAACTGAAAAAATCATTGACGCTTATTTCAGCCACACCATTCCTATTTATTGGGG
futA 67:20 (697) ACAAATTCAATCTGTGTTTTGAAAACACTCAAGGCTATGGCTATGTAACTGAAAAAATCATTGACGCTTATTTCAGCCACACCATTCCTATTTATTGGGG
futA 67:21 (693) ACAAATTCAATCTGTGTTTTGAAAACTCGCAAGGCTATGGCTATGTAACTGAAAAAATCATTGACGCTTATTTCAGCCATACCATTCCCATTTATTGGGG
futA 67:27 (697) ACAAATTCAATCTGTGTTTTGAAAACACTCAAGGCTATGGCTATGTAACTGAAAAAATCATTGACGCTTATTTCAGCCACACCATTCCTATTTATTGGGG
futB 67:18 (698) ACAAATTCAATCTGTGTTTTGAAAACTCGCAAGGCTATGGCTATGTAACTGAAAAAATCATTGACGCTTATTTCAGCCACACCATTCCTATTTATTGGGG
futB 67:19 (698) ACAAATTCAATCTGTGTTTTGAAAACACTCAAGGCTATGGCTATGTAACTGAAAAAATCATTGACGCTTATTTCAGCCACACCATTCCTATTTATTGGGG
futB 67:20 (692) ACAAATTCAATCTGTGTTTTGAAAACACTCAAGGCTATGGCTATGTAACTGAAAAAATCATTGACGCTTATTTCAGCCACACCATTCCTATTTATTGGGG
futB 67:21 (698) ACAAATTCAATCTGTGTTTTGAAAACTCGCAAGGCTATGGCTATGTAACTGAAAAAATCATTGACGCTTATTTCAGCCACACCATTCCTATTTATTGGGG
futB 67:27 (692) ACAAATTCAATCTGTGTTTTGAAAACACTCAAGGCTATGGCTATGTAACTGAAAAAATCATTGACGCTTATTTCAGCCACACCATTCCTATTTATTGGGG

(801)           801       810       820       830       840       850       860       870       880       890      900
futA 67:18 (795) GAGTCCTAGCGTGGCGAAAGATTTTAACCCTAAGAGTTTTGTGAATGTCCATGATTTCAAAAACTTTGATGAAGCGATTGATTATGTGAGATACTTGCAC
futA 67:19 (794) GAGTCCTAGCGTGGCGAAAGATTTTAACCCTAAGAGTTTTGTGAATGTCCATGATTTCAAAAACTTTGATGAAGCGATTGATTATGTGAGATACTTGCAC
futA 67:20 (797) GAGTCCTAGCGTGGCGAAAGATTTTAACCCTAAGAGTTTTGTGAATGTCCATGATTTCAAAAACTTTGATGAAGCGATTGATTATGTGAGATACTTGCAC
futA 67:21 (793) GAGTCCTAGCGTGGCGAAAGATTTTAACCCTAAGAGTTTTGTGAATGTCCATGATTTCAAAAACTTTGATGAAGCGATTGATTATGTGAGATACTTGCAC
futA 67:27 (797) GAGTCCTAGCGTGGCGAAAGATTTTAACCCTAAGAGTTTTGTGAATGTCCATGATTTCAAAAACTTTGATGAAGCGATTGATTATGTGAGATACTTGCAC
futB 67:18 (798) GAGTCCTAGCGTGGCGAAAGATTTTAACCCTAAGAGTTTTGTGAATGTCCATGATTTCAAAAACTTTGATGAAGCGATTGATTATGTGAGATACTTGCAC
futB 67:19 (798) GAGTCCTAGCGTGGCGAAAGATTTTAACCCTAAGAGTTTTGTGAATGTCCATGATTTCAAAAACTTTGATGAAGCGATTGATTATGTGAGATACTTGCAC
futB 67:20 (792) GAGTCCTAGCGTGGCGAAAGATTTTAACCCTAAGAGTTTTGTGAATGTCCATGATTTCAAAAACTTTGATGAAGCGATTGATTATGTGAGATACTTGCAC
futB 67:21 (798) GAGTCCTAGCGTGGCGAAAGATTTTAACCCTAAGAGTTTTGTGAATGTCCATGATTTCAAAAACTTTGATGAAGCGATTGATTATGTGAGATACTTGCAC
futB 67:27 (792) GAGTCCTAGCGTGGCGAAAGATTTTAACCCTAAGAGTTTTGTGAATGTCCATGATTTCAAAAACTTTGATGAAGCGATTGATTATGTGAGATACTTGCAC

(901)           901       910       920       930       940       950       960       970       980       990      1000
futA 67:18 (895) ACGCACAAAAACGCTTATTTAGACATGCTCTATGAAAACCCTTTAAACACCCTTGATGGGAAAGCTTACTTTTACCAAGATTTGAGTTTTAAAAAAATCC
futA 67:19 (894) ACGCACAAAAACGCTTATTTAGACATGCTCTATGAAAACCCTTTAAACACTATTGATGGGAAAGCTTACTTTTACCAAGATTTGAGTTTTAAAAAAATCC
futA 67:20 (897) ACGCACAAAAACGCTTATTTAGACATGCTCTATGAAAACCCTTTAAACACTATTGATGGGAAAGCTTACTTTTACCAAGATTTGAGTTTTAAAAAAATCC
futA 67:21 (893) ACGCACAAAAACGCTTATTTAGACATGCTCTATGAAAACCCTTTAAACACCCTTGATGGGAAAGCTTACTTTTACCAAGATTTGAGTTTTAAAAAAATCC
futA 67:27 (897) ACGCACAAAAACGCTTATTTAGACATGCTCTATGAAAACCCTTTAAACACTATTGATGGGAAAGCTTACTTTTACCAAGATTTGAGTTTTAAAAAAATCC
futB 67:18 (898) ACGCACAAAAACGCTTATTTAGACATGCTCTATGAAAACCCTTTAAACACCCTTGATGGGAAAGCTTACTTTTACCAAGATTTGAGTTTTAAAAAAATCC
futB 67:19 (898) ACGCACAAAAACGCTTATTTAGACATGCTCTATGAAAACCCTTTAAACACTATTGATGGGAAAGCTTACTTTTACCAAGATTTGAGTTTTAAAAAAATCC
futB 67:20 (892) ACGCACAAAAACGCTTATTTAGACATGCTCTATGAAAACCCTTTAAACACTATTGATGGGAAAGCTTACTTTTACCAAGATTTGAGTTTTAAAAAAATCC
futB 67:21 (898) ACGCACAAAAACGCTTATTTAGACATGCTCTATGAAAACCCTTTAAACACTATTGATGGGAAAGCTTACTTTTACCAAGATTTGAGTTTTAAAAAAATCC
futB 67:27 (892) ACGCACAAAAACGCTTATTTAGACATGCTCTATGAAAACCCTTTAAACACTATTGATGGGAAAGCTTACTTTTACCAAGATTTGAGTTTTAAAAAAATCC

(1001)          1001      1010      1020      1030      1040      1050      1060      1070      1080      1090     1100
futA 67:18 (995) TAGATTTTTTTAAAACGATTTTAGAAAACGACACGATCTATCACGATAACCCTTTCATTTTCTATCGTGATTTGAATGAGCCGTTAGCGACTATTGATGA
futA 67:19 (994) TAGATTTTTTTAAAACGATTTTAGAAAACGACACGATTTATCATAATAACCCTTTCATTTTCTATCGTGATTTGAATGAGCCTTTAGCGACTATTGATGA
futA 67:20 (997) TAGATTTTTTTAAAACGATTTTAGAAAACGACACGATTTATCATAATAACCCTTTCATTTTCTATCGTGATTTGAATGAGCCTTTAGCGACTATTGATGA
futA 67:21 (993) TAGATTTTTTTAAAACGATTTTAGAAAACGACACGATTTATCATAATAACCCTTTCATTTTCTATCGTGATTTGAATGAGCCGTTAGCGACTATTGATGA
futA 67:27 (997) TAGATTTTTTTAAAACGATTTTAGAAAACGACACGATTTATCATAATAACCCTTTCATTTTCTATCGTGATTTGAATGAGCCTTTAGCGACTATTGATGA
futB 67:18 (998) TAGATTTTTTTAAAACGATTTTAGAAAACGACACGATCTATCACGATAACCCTTTCATTTTCTATCGTGATTTGAATGAGCCGTTAGCGACTATTGATGA
futB 67:19 (998) TAGATTTTTTTAAAACGATTTTAGAAAACGACACGATTTATCATAATAACCCTTTCATTTTCTATCGTGATTTGAATGAGCCTTTAGCGACTATTGATGA
futB 67:20 (992) TAGATTTTTTTAAAACGATTTTAGAAAACGACACGATTTATCATAATAACCCTTTCATTTTCTATCGTGATTTGAATGAGCCTTTAGCGACTATTGATGA
futB 67:21 (998) TAGATTTTTTTAAAACGATTTTAGAAAACGACACGATTTATCATAATAACCCTTTCATTTTCTATCGTGATTTGAATGAGCCTTTAGCGACTATTGATGA
futB 67:27 (992) TAGATTTTTTTAAAACGATTTTAGAAAACGACACGATTTATCATAATAACCCTTTCATTTTCTATCGTGATTTGAATGAGCCTTTAGCGACTATTGATGA

(1101)          1101      1110      1120      1130      1140      1150      1160      1170      1180      1190     1200
futA 67:18(1095) TTTGAGGGTTAATTATGATGATTTGAGGGTTAATTATGATGATTTGAGGGTTAATTATGATGATTTGAGGGTTAATTATGATGATTTGAGAATCAATTAT
futA 67:19(1094) TTTGAGGGTTAATTATGATGATTTGAGAATCAATTAT---------------------------------------------------------------
futA 67:20(1097) TTTGAGGGTTAATTATGATGATTTGAGGGTTAATTATGATGATTTGAGGGTTAATTATGATGATTTGAGGGTTAATTATGATGATTTGAGGGTTAATTAT
futA 67:21(1093) TTTGAGGGTTAATTATGATGATTTGAGAATCAATTAT---------------------------------------------------------------
futA 67:27(1097) TTTGAGGGTTAATTATGATGATTTGAGGGTTAATTATGATGATTTGAGGGTTAATTATGATGATTTGAGGGTTAATTATGATGATTTGAGGGTTAATTAT
futB 67:18(1098) TTTGAGGGTTAATTATGATGATTTGAGGGTTAATTATGATGATTTGAGGGTTAATTATGATGATTTGAGGGTTAATTATGATGATTTGAGGGTTAATTAT
futB 67:19(1098) TTTGAGGGTTAATTATGATGATTTGAGGGTTAATTATGATGATTTGAGGGTTAATTATGATGATTTGAGGGTTAATTATGATGATTTGAGGGTTAATTAT
futB 67:20(1092) TTTGAGGGTTAATTATGATGATTTGAGGGTTAATTATGATGATTTGAGGGTTAATTATGATGATTTGAGGGTTAATTATGATGATTTGAGGGTTAATTAT
futB 67:21(1098) TTTGAGGGTTAATTATGATGATTTGAGGGTTAATTATGATGATTTGAGGGTTAATTATGATGATTTGAGGGTTAATTATGATGATTTGAGGGTTAATTAT
futB 67:27(1092) TTTGAGGGTTAATTATGATGATTTGAGGGTTAATTATGATGATTTGAGGGTTAATTATGATGATTTGAGGGTTAATTATGATGATTTGAGGGTTAATTAT
```

```
              (1201) 1201      1210      1220      1230      1240      1250      1260      1270      1280      1290      1300
futA 67:18(1195) ----------------------------------------------------------------------------------------------------
futA 67:19(1131) ----------------------------------------------------------------------------------------------------
futA 67:20(1197) GATGATTTGAGGGTTAATTATGATGATTTGAGGGTTAATTATGATGATTTGAGGGTTAATTATGATGATTTGAGGGTTAATTATGATGATTTGAGGGTTA
futA 67:21(1130) ----------------------------------------------------------------------------------------------------
futA 67:27(1197) GATGATTTGAGGGTTAATTATGATGATTTGAGGGTTAATTATGATGATTTGAGGGTTAATTATGATGATTTGAGGGTTAATTATGATGATTTGAGGGTTA
futB 67:18(1198) GATGATTTGAGGGTTAATTATGATGATTTGAGGGTTAATTAT----------------------------------------------------------
futB 67:19(1198) GATGATTTGAGGGTTAATTATGATGATTTGAGGGTTAATTATGATGATTTGAGGGTTAATTAT-------------------------------------
futB 67:20(1192) ----------------------------------------------------------------------------------------------------
futB 67:21(1198) GATGATTTGAGGGTTAATTATGATGATTTGAGGGTTAATTATGATGATTTGAGGGTTAATTAT-------------------------------------
futB 67:27(1192) ----------------------------------------------------------------------------------------------------

              (1301) 1301      1310      1320      1330      1340      1350      1360      1370      1380      1390      1400
futA 67:18(1195) ----------------------------------------------------------------------------------------------------
futA 67:19(1131) ----------------------------------------------------------------------------------------------------
futA 67:20(1297) ATTATGATGATTTGAGGGTTAATTATGATGATTTGAGGGTTAATTATGATGATTTGAGGGTTAATTATGATGATTTGAGGGTTAATTATGATGATTTGAG
futA 67:21(1130) ----------------------------------------------------------------------------------------------------
futA 67:27(1297) ATTATGATGATTTGAGGGTTAATTATGATGATTTGAGGGTTAATTATGATGATTTGAGGGTTAATTATGATGATTTGAGGGTTAATTATGATGATTTGAG
futB 67:18(1240) ----------------------------------------------------------------------------------------------------
futB 67:19(1261) ----------------------------------------------------------------------------------------------------
futB 67:20(1192) ----------------------------------------------------------------------------------------------------
futB 67:21(1261) ----------------------------------------------------------------------------------------------------
futB 67:27(1192) ----------------------------------------------------------------------------------------------------

              (1401) 1401      1410      1420      1430      1440      1450      1460      1470      1480      1490      1500
futA 67:18(1195) ----------------------------------------------------GAGCGCCTTTTGCAAAACGCTTCACCTTTATTGGAATTGTCCCAAAAC
futA 67:19(1131) ----------------------------------------------------GAGCGCCTTTTGCAAAACGCTTCACCTTTATTGGAATTGTCCCAAAAC
futA 67:20(1397) GGTTAATTATGATGATTTGAGGGTTAATTATGATGATTTGAGGGTTAATTATGACCGGCTTTTACAAAACGCCTCGCCTTTGTTGGAACTCTCTCAAAAC
futA 67:21(1130) ----------------------------------------------------GAGCGCCTTTTGCAAAACGCTTCACCTTTATTGGAATTGTCCCAAAAC
futA 67:27(1397) GGTTAATTAT------------------------------------------GACCGGCTTTTACAAAACGCCTCGCCTTTGTTGGAACTCTCTCAAAAC
futB 67:18(1240) ----------------------------------------------------GACCGGCTTTTACAAAACGCTTCACCTTTATTAGAACTCTCTCAAAAC
futB 67:19(1261) ----------------------------------------------------GACCGGCTTTTACAAAACGCTTCACCTTTATTAGAACTCTCTCAAAAC
futB 67:20(1192) ----------------------------------------------------GACCGGCTTTTACAAAACGCTTCACCTTTATTAAAACTCTCTCAAAAC
futB 67:21(1261) ----------------------------------------------------GACCGGCTTTTACAAAACGCTTCACCTTTATTAGAACTCTCTCAAAAC
futB 67:27(1192) ----------------------------------------------------GACCGGCTTTTACAAAACGCTTCACCTTTATTAAAACTCTCTCAAAAC

              (1501) 1501      1510      1520      1530      1540      1550      1560      1570      1580      1590
futA 67:18(1243) ACCTCTTTTAAAATCTATCGCAAAGCTTATCAAAAATCCTTACCCTTGTTGCGCGCCATAAGGAGATGGGTTAAGAAATTGGGTTTGTAA
futA 67:19(1179) ACCTCTTTTAAAATCTATCGCAAAGCTTATCAAAAATCCTTACCCTTGTTGCGCGCCATAAGGAGATGGGTTAAGAAATTGGGTTTGTAA
futA 67:20(1497) ACCACTTTTAAAATCTATCGCAAAGCCTATCAAAAATCCTTACCTTTGTTGCGTGCGGTGAGAAAGTTGATTAAAAAATTGGGTTTGTAA
futA 67:21(1178) ACCTCTTTTAAAATCTATCGCAAAGCTTATCAAAAATCCTTACCCTTGTTGCGCGCCATAAGGAGATGGGTTAAGAAATTGGGTTTGTAA
futA 67:27(1455) ACCACTTTTAAAATCTATCGCAAAGCCTATCAAAAATCCTTACCTTTGTTGCGTGCGGTGAGAAAGTTGATTAAAAAATTGGGTTTGTAA
futB 67:18(1288) ACCACTTTTAAAATCTATCGCAAAGCTTATCAAAAATCCTTACCTTTGTTGCGTGCCATAAGGAGATGGGTTAAAAAATAA---------
futB 67:19(1309) ACCACTTTTAAAATCTATCGCAAAGCTTATCAAAAATCCTTACCTTTGTTGCGTGCCATAAGGAGATGGGTTAAAAAATAA---------
futB 67:20(1240) ACCACTTTTAAAATCTATCGCAAAGCTTATCAAAAATCCTTACCTTTGTTGCGTGCCATAAGGAGATGGGTTAAAAAATAA---------
futB 67:21(1309) ACCACTTTTAAAATCTATCGCAAAGCTTATCAAAAATCCTTACCTTTGTTGCGTGCCATAAGGAGATGGGTTAAAAAATAA---------
futB 67:27(1240) ACCACTTTTAAAATCTATCGCAAAGCTTATCAAAAATCCTTACCTTTGTTGCGTGCCATAAGGAGATGGGTTAAAAAATAA---------
```
