# Supplementary material for: Lipopolysaccharide Diversity Evolving in Helicobacter pylori Communities through Genetic Modifications in Fucosyltransferases
Source: PLoS One. 2008 Nov 26;3(11):e3811. doi: 10.1371/journal.pone.0003811 (PMC2583950; doi:10.1371/journal.pone.0003811)
Supplement: Figure S3 — Amino acid sequence of FutA and FutB in five single-colony isolates. The DNA sequence from subisolates 67:18, 67:19, 67:20, 67:21 and 67:27 were translated into amino acid sequence. For comparisons of in-frame sequences, one C-residue was manually inserted or deleted in the C-tract of futA sequences of 67:19, 67:20, 67:21 and 67:27 before translation into protein sequence. A total of 43 amino acid positions were found to differ in at least one isolate. This calculation excludes the heptad-repeat sequence between positions 380–484 that is not shared by all isolates. (0.10 MB PDF) [file pone.0003811.s003.pdf]

```
                 (1) 1         10        20        30        40        50        60        70        80        90        100
futA 67:18       (1) MFQPLLDAFIESAPIKKKLPLNLPPPPLKIAVANWWGG--AEEFKKSTLYFILSQRYTITLHQNPNKPSDLVFGSPIGSARKILSYQNTKRVFHTGENEV
futA 67:19       (1) MFQPLLDAFIESAPIKKKLPLNLPPPPLKIAVANWWGG--AEEFKKSTLYFILSQRYTITLHQNPNKPSDLVFGSPIGSARKILSYQNTKRVFHTGENEV
futA 67:20       (1) MFQPLLDAFIESASIEKMASKSPPPP-LKIAVANWWRDKEVKEFKKSVLYFILSQRYTITLHQNPNKPSDLVFGNPIGAARKILSYQNTKRVFYTGENEV
futA 67:21       (1) MFQPLLDAFIESAPIKKKLPLNLPPP-LKIAVANWWRG--AEEFKKSTLYFILSQRYTITLHQNPNEPSDLVFGSPIGSARKILSYQNTKRVFYTGENEV
futA 67:27       (1) MFQPLLDAFIESASIEKMASKSPPPP-LKIAVANWWRDKEVKEFKKSVLYFILSQRYTITLHQNPNKPSDLVFGNPIGAARKILSYQNTKRVFYTGENEV
futB 67:18       (1) MFQPLLDAFIESASIEKMASKSPPPP-LKIAVANWWRDKEVKEFKKSVLYFILSQRYTITLHQNPNKPSDLVFGNPIGAARKILSYQNTKRVFYTGENEV
futB 67:19       (1) MFQPLLDAFIESASIEKMASKSPPPP-LKIAVANWWRDKEVKEFKKSVLYFILSQRYTITLHQNPNKPSDLVFGNPIGAARKILSYQNTKRVFYTGENEV
futB 67:20       (1) MFQPLLDAFIESASIEKMASKSPPPP-LKIAVANWWGG--AEEFKKSTLYFILSQRYTITLHQNPNKPSDLVFGNPIGAARKILSYQNTKRVFYTGENEV
futB 67:21       (1) MFQPLLDAFIESASIEKMASKSPPPP-LKIAVANWWRDKEVKEFKKSVLYFILSQRYTITLHQNPNKPSDLVFGNPIGAARKILSYQNTKRVFYTGENEV
futB 67:27       (1) MFQPLLDAFIESASIEKMASKSPPPP-LKIAVANWWGG--AEEFKKSTLYFILSQRYTITLHQNPNKPSDLVFGNPIGAARKILSYQNTKRVFYTGENEV

               (101) 101       110       120       130       140       150       160       170       180       190       200
futA 67:18 (99)      PNFNLFDYAIGFDELDFRDRYLRMPLYYDRLHHKAESVNDTTAPYKIKDNSLYTLKKPTHCFKENHPHLCAVVNNESDPLKRGFASFVASNPNAPKRNAF
futA 67:19 (99)      PNFNLFDYAIGFDELDFRDRYLRMPLYYDRLHHKAESVNDTTAPYKIKDNSLYTLKKPSHCFKENHPHLCAVVNNESDPLKRGFASFVASNPNAPKRNAF
futA 67:20(100)      PNFNLFDYAIGFDELDFRDRYLRMPLYYDRLHHKAESVNDTTAPYKIKDNSLYTLKKPTHCFKENHPHLCAVVNNESDPLKRGFASFVASNPNAPKRNAF
futA 67:21 (98)      PNFNLFDYAIGFDELDFRDRYLRMPLYYDRLHHKAESVNDTTAPYKIKDNSLYTLKKPSHHFKENHPHLCAVVNNESDPLKRGFASFVASNANAPMRNAF
futA 67:27(100)      PNFNLFDYAIGFDELDFRDRYLRMPLYYDRLHHKAESVNDTTAPYKIKDNSLYTLKKPTHCFKENHPHLCAVVNNESDPLKRGFASFVASNPNAPKRNAF
futB 67:18(100)      PNFNLFDYAIGFDELDFRDRYLRMPLYYDRLHHKAESVNDTTAPYKIKDNSLYTLKKPTHCFKENHPHLCAVVNNESDPLKRGFASFVASNPNAPKRNAF
futB 67:19(100)      PNFNLFDYAIGFDELDFRDRYLRMPLYYDRLHHKAESVNDTTAPYKIKDNSLYTLKKPSHCFKENHPHLCAVVNNESDPLKRGFASFVASNPNAPKRNAF
futB 67:20 (98)      PNFNLFDYAIGFDELDFRDRYLRMPLYYDRLHHKAESVNDTTAPYKIKSDSLYALKKPSHHFKENHPHLCAVVNDESDPLKRGFASFVASNPNAPKRNAF
futB 67:21(100)      PNFNLFDYAIGFDELDFRDRYLRMPLYYDRLHHKAESVNDTTAPYKIKDNSLYTLKKPTHCFKENHPHLCAVVNNESDPLKRGFASFVASNPNAPKRNAF
futB 67:27 (98)      PNFNLFDYAIGFDELDFRDRYLRMPLYYDRLHHKAESVNDTTAPYKIKSDSLYALKKPSHHFKENHPHLCAVVNDESDPLKRGFASFVASNPNAPKRNAF

               (201) 201       210       220       230       240       250       260       270       280       290       300
futA 67:18(199)      YEALNAIEPVTGGGSVKNTLGYNVKNKSEFLSQYKFNLCFENSQGYGYVTEKIIDAYFSHTIPIYWGSPSVAKDFNPKSFVNVHDFKNFDEAIDYVRYLH
futA 67:19(199)      YDALNSIEPVAGGGAVKNTLGYNVKNKSEFLSQYKFNLCFENTQGYGYVTEKIIDAYFSHTIPIYWGSPSVAKDFNPKSFVNVHDFKNFDEAIDYVRYLH
futA 67:20(200)      YDALNSIEPVAGGGAVKNTLGYNVKNKSEFLSQYKFNLCFENTQGYGYVTEKIIDAYFSHTIPIYWGSPSVAKDFNPKSFVNVHDFKNFDEAIDYVRYLH
futA 67:21(198)      YEALNAIEPVTGGGSVKNTLGYNVKNKSEFLSQYKFNLCFENSQGYGYVTEKIIDAYFSHTIPIYWGSPSVAKDFNPKSFVNVHDFKNFDEAIDYVRYLH
futA 67:27(200)      YDALNSIEPVAGGGAVKNTLGYNVKNKSEFLSQYKFNLCFENTQGYGYVTEKIIDAYFSHTIPIYWGSPSVAKDFNPKSFVNVHDFKNFDEAIDYVRYLH
futB 67:18(200)      YEALNAIEPVTGGGSVKNTLGYNVKNKSEFLSQYKFNLCFENSQGYGYVTEKIIDAYFSHTIPIYWGSPSVAKDFNPKSFVNVHDFKNFDEAIDYVRYLH
futB 67:19(200)      YDALNSIEPVAGGGAVKNTLGYNVKNKSEFLSQYKFNLCFENTQGYGYVTEKIIDAYFSHTIPIYWGSPSVAKDFNPKSFVNVHDFKNFDEAIDYVRYLH
futB 67:20(198)      YDALNSIEPVAGGGAVKNTLGYNVKNKSEFLSQYKFNLCFENTQGYGYVTEKIIDAYFSHTIPIYWGSPSVAKDFNPKSFVNVHDFKNFDEAIDYVRYLH
futB 67:21(200)      YEALNAIEPVTGGGSVKNTLGYNVKNKSEFLSQYKFNLCFENSQGYGYVTEKIIDAYFSHTIPIYWGSPSVAKDFNPKSFVNVHDFKNFDEAIDYVRYLH
futB 67:27(198)      YDALNSIEPVAGGGAVKNTLGYNVKNKSEFLSQYKFNLCFENTQGYGYVTEKIIDAYFSHTIPIYWGSPSVAKDFNPKSFVNVHDFKNFDEAIDYVRYLH

               (301) 301       310       320       330       340       350       360       370       380       390       400
futA 67:18(299)      THKNAYLDMLYENPLNTLDGKAYFYQDLSFKKILDFFKTILENDTIYHDNPFIFYRDLNEPLATIDDLRVNYDDLRVNYDDLRVNYDDLRVNYDDLRINY
futA 67:19(299)      THKNAYLDMLYENPLNTIDGKAYFYQDLSFKKILDFFKTILENDTIYHNNPFIFYRDLNEPLATIDDLRVNYDDLRINY---------------------
futA 67:20(300)      THKNAYLDMLYENPLNTIDGKAYFYQDLSFKKILDFFKTILENDTIYHNNPFIFYRDLNEPLATIDDLRVNYDDLRVNYDDLRVNYDDLRVNYDDLRVNY
futA 67:21(298)      THKNAYLDMLYENPLNTLDGKAYFYQDLSFKKILDFFKTILENDTIYHNNPFIFYRDLNEPLATIDDLRVNYDDLRINY---------------------
futA 67:27(300)      THKNAYLDMLYENPLNTIDGKAYFYQDLSFKKILDFFKTILENDTIYHNNPFIFYRDLNEPLATIDDLRVNYDDLRVNYDDLRVNYDDLRVNYDDLRVNY
futB 67:18(300)      THKNAYLDMLYENPLNTLDGKAYFYQDLSFKKILDFFKTILENDTIYHDNPFIFYRDLNEPLATIDDLRVNYDDLRVNYDDLRVNYDDLRVNYDDLRVNY
futB 67:19(300)      THKNAYLDMLYENPLNTIDGKAYFYQDLSFKKILDFFKTILENDTIYHNNPFIFYRDLNEPLATIDDLRVNYDDLRVNYDDLRVNYDDLRVNYDDLRVNY
futB 67:20(298)      THKNAYLDMLYENPLNTIDGKAYFYQDLSFKKILDFFKTILENDTIYHNNPFIFYRDLNEPLATIDDLRVNYDDLRVNYDDLRVNYDDLRVNYDDLRVNY
futB 67:21(300)      THKNAYLDMLYENPLNTIDGKAYFYQDLSFKKILDFFKTILENDTIYHNNPFIFYRDLNEPLATIDDLRVNYDDLRVNYDDLRVNYDDLRVNYDDLRVNY
futB 67:27(298)      THKNAYLDMLYENPLNTIDGKAYFYQDLSFKKILDFFKTILENDTIYHNNPFIFYRDLNEPLATIDDLRVNYDDLRVNYDDLRVNYDDLRVNYDDLRVNY

               (401) 401       410       420       430       440       450       460       470       480       490       500
futA 67:18(399)      ------------------------------------------------------------------------------------ERLLQNASPLLELSQN
futA 67:19(378)      ------------------------------------------------------------------------------------ERLLQNASPLLELSQN
futA 67:20(400)      DDLRVNYDDLRVNYDDLRVNYDDLRVNYDDLRVNYDDLRVNYDDLRVNYDDLRVNYDDLRVNYDDLRVNYDDLRVNYDDLRVNYDRLLQNASPLLELSQN
futA 67:21(377)      ------------------------------------------------------------------------------------ERLLQNASPLLELSQN
futA 67:27(400)      DDLRVNYDDLRVNYDDLRVNYDDLRVNYDDLRVNYDDLRVNYDDLRVNYDDLRVNYDDLRVNYDDLRVNY--------------DRLLQNASPLLELSQN
futB 67:18(400)      DDLRVNYDDLRVNY----------------------------------------------------------------------DRLLQNASPLLELSQN
futB 67:19(400)      DDLRVNYDDLRVNYDDLRVNY---------------------------------------------------------------DRLLQNASPLLELSQN
futB 67:20(398)      ------------------------------------------------------------------------------------DRLLQNASPLLKLSQN
futB 67:21(400)      DDLRVNYDDLRVNYDDLRVNY---------------------------------------------------------------DRLLQNASPLLELSQN
futB 67:27(398)      ------------------------------------------------------------------------------------DRLLQNASPLLKLSQN

               (501) 501       510                529
futA 67:18(415)      TSFKIYRKAYQKSLPLLRAIRRWVKKLGL
futA 67:19(394)      TSFKIYRKAYQKSLPLLRAIRRWVKKLGL
futA 67:20(500)      TTFKIYRKAYQKSLPLLRAVRKLIKKLGL
futA 67:21(393)      TSFKIYRKAYQKSLPLLRAIRRWVKKLGL
futA 67:27(486)      TTFKIYRKAYQKSLPLLRAVRKLIKKLGL
futB 67:18(430)      TTFKIYRKAYQKSLPLLRAIRRWVKK---
futB 67:19(437)      TTFKIYRKAYQKSLPLLRAIRRWVKK---
futB 67:20(414)      TTFKIYRKAYQKSLPLLRAIRRWVKK---
futB 67:21(437)      TTFKIYRKAYQKSLPLLRAIRRWVKK---
futB 67:27(414)      TTFKIYRKAYQKSLPLLRAIRRWVKK---
```
