# Supplementary material for: Lipopolysaccharide Diversity Evolving in Helicobacter pylori Communities through Genetic Modifications in Fucosyltransferases
Source: PLoS One. 2008 Nov 26;3(11):e3811. doi: 10.1371/journal.pone.0003811 (PMC2583950; doi:10.1371/journal.pone.0003811)
Supplement: Table S1 — Primers used in this study (0.05 MB PDF) [file pone.0003811.s004.pdf]

Table S1: Primers used in this study

Primer name	Sequence 5′–3′	Target gene/region[1]	Reference
cagAsbra F	ATG ATG GCG TGA TGT TTG T	*cagA*	[1]
cagAsbra R	TTT TCA AGG TCG CTT TTT GC	*cagA*	[1]
UpCagF	ACT TTC ACG CCC TTT CCC TCC	*cag* PAI empty site	[1]
DownCagR	TTG CAT GCG TTA TTA TTT CAC	*cag* PAI empty site	[1]
HP0379 F	CTC TCG TGA TCT TGG CTT ATT	*futA* upstream	[2]
HP0379 R	AAG TAG CGT CTG CGA TGA	*futA* downstream	[2]
C-tract up 379	CTT GGC TTA TTT CAA ACG C	*futA* upstream	[2]
C-tract up 651	GCC CTA ATC AAG CCT TTG	*futB* upstream	[2]
C-tract down 3	CCG GTG TAA AAC ACT CGT TTA G	*futA, futB*	[2]
C-tract down 4	TCA TCA AAG CCT ATG GCG TA	*futA, futB*	[2]
FucT F1	TTC CAA CCC CTA TTA GAC G	*futA, futB*	This study
FucT F2	CAA TCA CCC TCC ACC AAA AC	*futA, futB*	This study
FucT F3	GAT CGT TAT TTG AGA ATG CC	*futA, futB*	This study
FucT F4	CAA AGA CAA CAG CCT TTA TGC	*futA, futB*	This study
FucT F5	GCG AGT TTT TAA GCC AAT AC	*futA, futB*	This study
FucT R1	GTT AAA ATC TTT CGC CAC G	*futA, futB*	This study
FucT R2	GGG TGT TTA AAG GGT TTT C	*futA, futB*	This study
FucT R3	TTT CTC ACA CTT CCT CCC C	*futA, futB*	This study
FucT R4	GTT GCT CGC TAC AAA ACT G	*futA, futB*	This study
Repeat up-2	CGC ACC CAA ACG CTT ATT TA	*futA, futB*	[2]
Repeat up-3	CGA TTT TAG AAA ACG ACA CGA	*futA, futB*	This study
Repeat down 379	GAT GAT AGC GCA AGG GGT TT	*futA* downstream	[2]
Repeat down 651	AAA ACC CCA CGC TCA AAA A	*futB* downstream	[2]
379 down R1	GGC CAA TAT CGC TGG TTT TA	*futA* downstream	This study
379 down R2	AAG TGG TGG ATG CGA AAT TG	*futA* downstream	This study
379 down R3	TGG AGT TTT GAA GTG GTG GA	*futA* downstream	This study
flaB:F	AAG GCA TGC TCG CTA GCG	*flaB*	[3]
flaB:R	TAA TGT CTC TAG CGT CGG	*flaB*	[3]
recAF	GAA ATT TAT GGG CAG AGT C	*recA*	[4]
recAR	GAT AAA AAT GAG AGT GGT GTT	*recA*	[4]
Hp71as	GGC AAT GCT AGG ACT TGT	*ureI*	[3]
Hp71S3	TCC CTT AGA TTG CCA ACT AAA CGC	*ureI*	[3]
16SF	TGG CAA TCA GCG TCA GGT AAT G	16S rRNA	[4]
16SR	GCT AAG AGA TCA GCC TAT GTC C	16S rRNA	[4]

[1]Upstream and downstream indicate that the primer binding sequence is located outside the coding region of the genes.

References:

1. Nilsson C, Sillén A, Eriksson L, Strand M-L, Enroth H, et al. (2003) Correlation between *cag* pathogenicity island composition and *Helicobacter pylori*-associated gastroduodenal disease. Infect Immun 71: 6573-6581.
2. Nilsson C, Skoglund A, Moran AP, Annuk H, Engstrand L, et al. (2006) An enzymatic ruler modulates Lewis antigen glycosylation of *Helicobacter pylori* LPS during persistent infection. Proc Natl Acad Sci USA 103: 2863-2868.
3. Lundin A, Björkholm B, Kupershmidt I, Unemo M, Nilsson P, et al. (2005) Slow genetic divergence of *Helicobacter pylori* strains during long-term colonization. Infect Immun 73: 4818-4822.
4. Björkholm B, Lundin A, Sillén A, Guillemin K, Salama N, et al. (2001) Comparison of genetic divergence and fitness between two subclones of *Helicobacter pylori*. Infect Immun 69: 7832-7838.
